# Dissecting myocardial and systemic drivers of cardiac dysfunction in the murine CVB3 myocarditis model using microRNA-guided viral detargeting

**DOI:** 10.1007/s00395-026-01187-4

**Published:** 2026-05-29

**Authors:** Sarah Ochs, Sandra Pinkert, Sophia Borowski, Lisa Gerarda Maria Huis in ’t Veld, Nicolas Kelm, Anja Geisler, Henry Fechner, Ziya Kaya, Anne Hausen, Karin Klingel, Matthias Martin Gaida, Antje Beling

**Affiliations:** 1 Institute of Biochemistry, Charité – Universitätsmedizin Berlin, corporate member of Freie Universität Berlin and Humboldt-Universität zu Berlin, Charitéplatz 1, 10117 Berlin, Germany; 2 Deutsches Zentrum Für Herz-Kreislauf-Forschung, partner site Berlin, 10117 Berlin, Germany; 3 Cardiopathology, Institute for Pathology and Neuropathology, University Hospital Tübingen, 72076 Tübingen, Germany; 4 Department of Applied Biochemistry, Institute of Biotechnology, Technische Universität Berlin, 15533 Berlin, Germany; 5 Kardiologie, Angiologie und Pneumologie, Medizinische Klinik Für Innere Medizin III, Universitätsklinikum Heidelberg, 69120 Heidelberg, Germany; 6 Deutsches Zentrum für Herz-Kreislauf-Forschung (DZHK), partner site Heidelberg, 69120 Heidelberg, Germany; 7 Institute of Pathology, University Medical Center Mainz, Johannes-Gutenberg-Universität Mainz, 55131 Mainz, Germany; 8 TRON, Translational Oncology at the University Medical Center, Johannes Gutenberg University Mainz, 55131 Mainz, Germany; 9 Research Center for Immunotherapy, University Medical Center Mainz, Johannes-Gutenberg-Universität Mainz, 55131 Mainz, Germany; 10 Department of Medicine I, Department of Gastroenterology and Hepatology, Faculty of Medicine, University Hospital Carl Gustav Carus, TUD Dresden University of Technology, 01069 Dresden, Germany

**Keywords:** Infection, Inflammation, Viral myocarditis, microRNA targeting, Cardiac dysfunction, Coxsackievirus B3

## Abstract

In murine coxsackievirus B3 myocarditis, the most widely used experimental model of viral myocarditis, functional alterations such as reduced cardiac output are frequently interpreted as surrogates of myocardial injury and inflammation, despite concurrent systemic illness. The relative contribution of systemic disease processes to these functional changes remains poorly defined. We therefore aimed to disentangle myocardial and systemic drivers of cardiac dysfunction using a microRNA-guided viral detargeting approach. We engineered a cardiomyocyte-detargeted virus by inserting target sequences for the muscle-enriched microRNA-1 into the viral genome, selectively suppressing viral replication in cardiac muscle while largely preserving systemic infection. This enabled direct comparison between conventional myocarditis and cardiac-attenuated infection in mice, using serial echocardiography as the primary non-invasive functional readout of cardiac performance. Day 3 post infection was analyzed as the acute phase and day 7 as a later stage associated with peak myocardial inflammation. Cardiac viral titers were profoundly reduced by detargeting (approximately 3-log at day 3 and 6–7-log at day 7), accompanied by absence of troponin T release, inflammatory cell infiltration, and histological myocarditis. Despite this, cardiac output was reduced at day 3 and remained impaired at day 7 in both groups. This reduction was associated with decreased left ventricular end-diastolic volume, while ejection fraction remained preserved. At day 3, additional reductions in cardiac output and global longitudinal strain were observed in control-virus-infected mice. At day 7, cardiac output remained comparably reduced despite marked differences in myocardial injury. These findings demonstrate that systemic infection substantially contributes to impaired cardiac function and that echocardiographic readouts reflect a composite phenotype. Functional alterations should therefore be interpreted in the context of systemic effects and integrated with cross-organ analyses to enable accurate interpretation in preclinical myocarditis models.

**Graphical Abstract:**

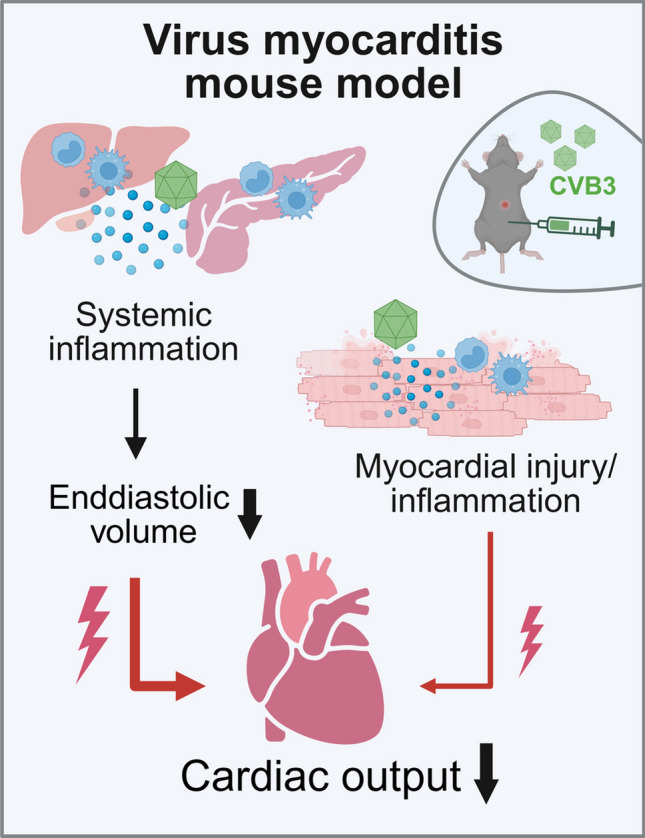

**Supplementary Information:**

The online version contains supplementary material available at 10.1007/s00395-026-01187-4.

## Introduction

Myocarditis is an inflammatory disease of the heart with diverse etiologies and outcomes. Viral infections remain the most frequent trigger, and other causes include autoimmune diseases, drug reactions, toxins, and systemic inflammatory syndromes. Clinical presentation ranges from isolated chest pain and dyspnea to fulminant cardiogenic shock and sudden cardiac arrest [43]. While acute myocarditis may resolve completely, progression to chronic myocarditis with persistent low-grade inflammation and fibrotic remodeling can lead to dilated cardiomyopathy (DCM), a leading cause of heart failure in Western countries [30]. Diagnosis of acute myocarditis integrates clinical presentation with laboratory and imaging findings. Initial evaluation includes electrocardiography, cardiac biomarkers, and inflammatory markers [8, 12]. Transthoracic echocardiography (TTE) serves as the first-line imaging modality, assessing systolic function, and pericardial effusion, with global longitudinal strain (GLS) providing enhanced sensitivity for early dysfunction [8, 18]. The ESC 2025 guidelines classify echocardiographic dysfunction as supportive but not diagnostic [46]. Cardiac magnetic resonance (CMR) is instead recommended as the key non-invasive modality, using updated Lake Louise criteria combining oedema and injury markers [11, 12]. Endomyocardial biopsy (EMB) remains essential in high-risk cases, enabling histological characterization and viral genome detection [30, 46].

Given the scarcity of human biopsy material from early-stage myocarditis, murine models are indispensable for understanding the temporal relationship between viral infection, inflammation, and cardiac dysfunction. The Coxsackievirus B3 (CVB3) mouse model is the most widely used experimental system and has shaped much of our current understanding of viral myocarditis [27]. After intraperitoneal inoculation, CVB3 infection exhibits a multiphasic course, with dominant systemic involvement at day 3 and cardiac viral genomes detectable before inflammatory infiltrates emerge [9, 22, 27]. This phase features systemic [22, 24], but also myocardial interferon and cytokine induction [2, 20], demonstrating that antiviral and inflammatory signaling precede histologically apparent myocardial necrosis and leukocyte infiltration. By the subacute phase (days 7–8), myocardial involvement becomes overt with dense immune cell infiltration defining histological myocarditis [3, 27]. In this viral myocarditis mouse model, cardiac function was predominantly assessed by echocardiography [51, 52] as the primary non-invasive functional readout, enabling longitudinal assessment in infected animals, or invasive conductance catheterization [36, 40, 48] at this subacute phase. Nowadays, available advanced imaging modalities and invasive hemodynamic measurements are often limited in this setting by feasibility and animal welfare considerations. Functional impairment has largely been interpreted as a consequence of overt myocarditis [36, 40, 48, 51, 52]. However, our previous work demonstrated that marked reductions in cardiac output already occur during the acute phase, before myocarditis becomes detectable by classical diagnostic criteria [22, 25]. These observations indicate that cardiac dysfunction can arise in the context of systemic infection, preceding overt myocardial inflammation. Consequently, it remains unclear to what extent imaging-based functional alterations in this model reflect direct myocardial injury versus systemic disease processes or immune-mediated effects. Importantly, this distinction cannot be resolved using conventional experimental approaches, in which systemic infection and cardiac involvement occur simultaneously, and therefore remains insufficiently defined. This limitation is particularly relevant given the widespread reliance on echocardiography as a primary non-invasive functional readout in preclinical myocarditis studies, aligned with the principles of reduction and refinement in animal research.

To directly address this unresolved issue, we sought an experimental strategy that allows selective attenuation of CVB3 replication in the heart while preserving systemic infection. MicroRNA-based targeting has emerged as a robust approach to achieve tissue-selective regulation of viral replication and transgene expression across multiple systems [6]. In the CVB3 model, proof-of-concept studies demonstrated that insertion of pancreas-specific miR-375 target sites efficiently suppresses viral replication in the exocrine pancreas and prevents the severe pancreatitis that otherwise dominates the murine disease phenotype [38]. Consistent with this concept, targeting muscle-enriched microRNAs such as miR-133 or miR-206 attenuated cardiac replication and virulence in mice, further supporting the feasibility of tissue-specific viral control [15]. More recently, combined insertion of miR-375 and miR-1 target sites completely abolished viral replication in both pancreas and heart while preserving oncolytic efficacy in tumor models [14]. Building on these prior observations, we here employed a CVB3 variant carrying isolated miR-1 target sites (CVB3-1), alongside a control virus encoding a non-mammalian miR-39 target site (CVB3-39). Our strategy permits systemic infection with preserved replication in non-muscle organs, while selectively attenuating replication in the heart. Using this experimental system, we aimed to dissect how viral myocarditis modulates echocardiographic measures of cardiac function during CVB3 infection, thereby enabling a direct experimental separation of myocardial and systemic contributions, rather than assuming a direct equivalence between functional impairment and myocardial inflammation.

## Material and methods

### Animals

C57BL/6J male mice (6–7 weeks of age) were obtained from the Charité FEM breeding facility and were infected intraperitoneally (i.p.) with 10^5^ pfu/mouse of either CVB3-1 or CVB3-39. Animals were housed under standard conditions with ad libitum access to food and water. Body weight was measured daily. Mice were euthanized by continuous inhalation with an overdose of isoflurane (approximately 15% vapor concentration generated in a sealed chamber using the drop method) (CP-Pharma, Burgdorf, Germany) on days 0, 3 or 7 post-infections (p.i.). Prior to organ collection, transcardiac perfusion with PBS (Biochrom, Holliston, Massachusetts, USA) was performed. Organs were removed, weighed and tissue pieces were processed for histology or snap-frozen in liquid nitrogen and then stored at -80 °C.

All animal experiments were approved by the local animal welfare authorities in Berlin (LAGeSo; registration number G0119/20, T-CH0007-24, T0032/07) in accordance with the German Animal Welfare Act, the Animal Welfare Laboratory Regulations, and the European Parliament Directive 2010/63/EU on the protection of animals used for scientific purposes. Additionally, 0.1 mg/mL Tramal (tramadol hydrochloride; Grünenthal GmbH, Aachen, Germany) was administered orally via drinking water ad libitum as shown before [37]. Animal welfare was checked at least twice daily.

### Viruses

The plasmid pMKS1-H3N-miR-1(3x)TS was generated using the In-Fusion HD Cloning Kit (Takara Bio USA, Mountain View, CA, USA). For this construct, the plasmid pMKS1-H3N-miR-375(2x)-miR-1(2x)TS [14] was linearized using the primers IF 3xmiR-1 s (5´-GCG CAT ACA TAC TTC TTT ACA TTC C-3´) and IF 3xmiR-1 as (5´-GAA GTA TGT ATG CGC TGG AAT GTA AAG AAG TAT GTA TGG TGC CTA TCC TCG AGC G-3´), followed by In-Fusion assembly according to the manufacturer’s instructions. For generation of CVB3-208, three tandem copies of the miR-208 target sequence were inserted into the 3′ untranslated region of the CVB3 genome using the In-Fusion HD Cloning Kit (Takara Bio, USA). The following primers were used: Destination primer forward (5’-CCTCGAGGGGGGGCCCGG-3'), destination primer reverse (5’-TTTAGAAAGAGTCCAACCACTTCC-3'), insert primer forward (5’-TGGACTCTTTCTAAAACGCAGGAAGTGGTTGGACTC-3'), insert primer reverse (5’-GGCCCCCCCTCGAGGAGCCAATCTAAATTATTTCAAATTG-3'). All primers were designed with the In-Fusion online primer design tool (Takara Bio, USA). The construction of pMKS1-H3N-39(3 ×)TS has been reported elsewhere [38]. The plasmids were transfected into HEK293T cells using polyethylenimine (PEI; Polysciences, Warrington, PA, USA) to generate infectious viral particles. All viral stocks were subsequently amplified by a single passage in HeLa cells.

### Echocardiography

Transthoracic echocardiography was performed to assess cardiac function in C57BL/6J mice at baseline (day 0, one day prior to infection) and on days 3 and 7 post infection, prior to sacrifice. Mice were anesthetized by continuous inhalation of isoflurane (CP-Pharma, Burgdorf, Germany) (3–5% for induction, 1.5–2% for maintenance), shaved, and positioned on a heated imaging platform equipped with real-time monitoring of body temperature, respiration, electrocardiogram, and heart rate. Echocardiographic imaging was performed using a Vevo 3100 high-resolution ultrasound system (FUJIFILM VisualSonics, Toronto, ON, Canada) equipped with an MX400 transducer mounted on an adjustable holder and positioned on the thoracic wall using ultrasound gel. Image acquisition was conducted under continuous physiological monitoring to ensure stable anesthesia and animal well-being throughout the procedure. Standardized B-mode recordings were obtained in parasternal long-axis and mid-papillary short-axis views. M-mode measurements were performed in the mid-papillary short-axis. The apical four-chamber view was used to acquire pulsed-wave Doppler signals of transmitral inflow and tissue Doppler signals of the septal mitral annulus. Pulsed-wave Doppler measurements yielded peak transmitral flow velocities during early (MV E) and atrial (MV A) diastolic filling, as well as isovolumetric relaxation time (IVRT), isovolumetric contraction time (IVCT), and aortic ejection time (AET). Tissue Doppler imaging was used to assess peak septal mitral annulus velocities during early diastole (MV E′), atrial contraction (MV A′), and systole (MV S′). Left ventricular (LV) end-diastolic and end-systolic volumes (LVEDV, LVESV), stroke volume (SV), cardiac output (CO), and ejection fraction (EF) were derived from endocardial border tracking. Left ventricular anterior (LVAW;d) and posterior (LVPW;d) wall thicknesses as well as left ventricular internal diameter in diastole (LVID;d) were quantified from M-mode tracings, and myocardial mass (LVmass) was calculated using the standard geometric formula. For speckle-tracking analysis, at least three independent recordings from parasternal long-views were analyzed per mouse. Global longitudinal strain (GLS) was derived. Derived indices included the E/A and E/E′. All recordings were digitally stored and analyzed offline using VevoLab software version 5.7.1 (FUJIFILM VisualSonics, Amsterdam, The Netherlands). All image acquisition and analysis procedures were performed in accordance with current guidelines for small-animal echocardiography [4, 53].

### Serum parameters

Serum was separated from whole blood samples which were collected via retrobulbar blood sampling. Separation was performed by centrifugation (10.000 × g, 10 min) after 30 min incubation at RT. Serum aliquots were stored at -80 °C until analysis. Serum concentrations of bile acids, aspartate aminotransferase (AST), alanine aminotransferase (ALT), albumin, lipase and lactate dehydrogenase (LDH) were commercially analyzed by ANTECH™ Diagnostics (Berlin, Germany). Given that elevated bile acid levels, even in naïve mice, may confound metabolic and cardiovascular readouts [13], animals with bile acid concentrations exceeding twofold the group mean were predefined as outliers and excluded from all analyses. Serum was additionally analyzed with the LEGENDplex™ Mouse Anti-Virus Response Panel (13-plex) (Biolegend, San Diego, California, USA) according to manufacturer's protocol, fixed with 2% ROTI^TM^Histofix (Carl Roth, Karlsruhe, Germany) in PBS for 30 min at RT and analyzed via flow cytometry with a BD FACSymphony™ A3 cell analyzer (BD, Franklin Lakes, New Jersey, USA). Data analysis was performed with the LEGENDplex™ Qognit cloud-based software (BioLegend). For high sensitivity cardiac troponin T (hsTNT) quantification, samples were diluted 1:17.5 in 0.9% NaCl (B. Braun, Melsungen, Germany) and tested with an electrochemiluminescence immunoassay (ECLIA) analyzer (Elecsys 2010 Analyzer, Roche Diagnostics, Mannheim, Germany).

### Histology

Tissue samples for histology were transferred into 4% ROTI^TM^Histofix in PBS and fixed followed by embedding in paraffin, sectioning and hematoxylin and eosin (H&E) staining. The slides were digitized with a NanoZoomer 2.0‐HT scanner (Hamamatsu Photonics, Herrsching, Germany). Evaluation was made using the corresponding software NDP.view2 (Hamamatsu). Myocardial inflammation was assessed with a myocarditis scoring system ranging from 0 to 4 [49] and pancreas destruction was scored ranging from 0 to 100% as was described elsewhere. Animals lacking complete pancreatic destruction at endpoints (day 3 or 7 p.i.) were classified as non-responders and excluded from further analysis. Necrosis and inflammation in the liver sections were scored using an established histopathological score [50]. Myositis in skeletal muscle was evaluated using two complementary scoring systems. First, inflammatory cell infiltration was graded on a scale of 0–3, where 0 indicates no infiltration, 1 represents mild infiltration, 2 denotes moderate infiltration, and 3 signifies severe infiltration [44]. Second, the muscle inflammation score (MIS) was applied to assess muscle fiber necrosis within individual muscle blocks [1]. The MIS grading is as follows: Grade 0 – no necrotic fibers; Grade 1 – up to 5 necrotic fibers per lesion; Grade 2 – 5 to 30 necrotic fibers; Grade 3 – more than 30 necrotic fibers; and Grade 4 – diffuse, extensive necrosis. When multiple lesions of the same grade were present within a single muscle section, 0.5 points were added to the score. All scoring has been performed by blinded pathologists.

### Cell culture experiments

HeLa cells (ATCC CCL-2) were cultured in minimum essential medium (MEM; Thermo Fisher Scientific, Carlsbad, California, USA) supplemented with 5% fetal calf serum (FCS) (Sigma Aldrich, Burlington, Massachusetts, USA), 1% penicillin/streptomycin, 1% non-essential amino acid (NEAA; all Gibco/Life Technologies, Carlsbad, California, USA) and 20 mM HEPES at 37 °C in a humidified atmosphere containing 5% CO₂ and passaged three times per week. HEK293T cells (kindly supplied by Henry Fechner) were cultured in Dulbecco’s modified Eagle’s medium (DMEM; Gibco/Life Technologies, Carlsbad, California, USA) supplemented with 10% FCS, 1% penicillin/streptomycin, 1 mM sodium pyruvate (Gibco/Life Technologies, Carlsbad, California, USA). Cells were maintained under identical incubation conditions and passaged three times per week. For experiments using primary embryonic cardiomyocytes, female C57BL/6J mice were bred at the Charité FEM breeding facility to obtain embryos, from which embryonic cardiomyocytes were isolated at embryonic day 14.5 (E14.5), as previously described [35].

Cells were infected with CVB3-39, CVB3-1, or CVB3-208 at a multiplicity of infection (MOI) of 1 or 5 for 30 min. Following infection, the inoculum was removed and replaced with fresh culture medium. Cells were harvested at 0, 8, or 24 h post infection for plaque assays, RT–qPCR, or western blot analysis. For plaque assays, cells were lysed by three freeze–thaw cycles. For western blot analysis, cells were washed once with PBS and lysed in RIPA buffer (Thermo Fisher Scientific, Waltham, MA, USA) supplemented with 1 × cOmplete protease inhibitor cocktail (Roche), snap-frozen, and stored at − 80 °C until further processing. For RT–qPCR, cells were harvested in TRIzol reagent (Thermo Fisher Scientific), snap-frozen, and stored at − 80 °C.

### Quantification of virus titer

Viral titers in mouse tissues and cell lysates were determined by plaque assay on HeLa cells as previously described [37]. Serial tenfold dilutions of tissue homogenates or cell lysates were prepared, and plaque assays were performed in duplicate. Cells were overlaid with Eagle agar and incubated for 2 days. Plaques were then visualized by staining and counted after an additional 2–4 h incubation.

### RNA-Isolation and quantitative Reverse Transcription PCR (RT-qPCR)

RNA-Isolation and RT-qPCR was performed as previously described [47] with specific primers and probes for *Ifit3, Ifn-β, Il-1β, Tnf-α, Ccl2, Cxcl10* using TaqMan gene expression assays (Thermo fisher scientific, Waltham, Massachusetts, USA). Additionally, the following primers were used: murine *Hprt* (forward primer: 5’—ATC ATT ATG CCG AGG ATT TGG AA – 3’; reverse primer: 3’—TTG AGC ACA CAG AGG GCC A – 5’; probe: 5’ – FAM-TGG ACA GGA CTG AAA GAC TTG CTC GAG ATG – 3’TAMRA) and CVB3 (forward primer: 5’ – CCC TGA ATG CGG CTA ATCC– 3’; reverse primer: 3’ – ATT GTC ACC ATA AGC AGC CA – 5’; probe: 5’ – FAM-TGCAGCGGAACCG-MGB – 3’). For relative quantification with the ΔC(t) method, *Hprt* was used as an endogenous expression control.

### miRNA expression analysis

Cell lines and organ pieces were analyzed for expression of miR-1, miR-208 and U6 snRNA as endogenous expression control using TaqMan™ MicroRNA Assay and TaqMan™ Micro-RNA Control Assay (Thermo fisher scientific, Waltham, Massachusetts, USA). Therefore, RNA was isolated with TRIzol as described before. Then, cDNA was synthesized using 1 ng/µl RNA and RT-qPCR was performed according to the manufacturer’s protocol.

### Western blot analysis

Cell pellets were lysed in lysis buffer for 20 min and then centrifuged at 16.000 × g for 10 min at 4 °C. Protein concentration was determined with Bradford assay (Thermo Fisher Scientific, Waltham, MA, USA) according to manufacturer’s protocol and samples were adjusted to a final concentration of 1 µg/ml. Subsequently, 6 × sample buffer (375 mM Tris–HCl, pH 6.8; 12% SDS; 0.6 M DTT; 60% glycerol; 0.03% bromophenol blue; all in Milli-Q water) was added, and proteins were denatured at 95 °C for 5 min. Protein lysates were then loaded on 15% SDS-PAGE gels and transferred onto 0.22 µm nitrocellulose membranes (Odyssey Nitrocellulose Membrane; LI-COR Biosciences, Lincoln, NE, USA). Membranes were dried, blocked for 1 h in 1 × Rotiblock (Carl ROTH, Karlsruhe, Germany) and incubated overnight with primary antibodies against VP1 (clone 31A2, Mediagnost, Reutlingen, Germany; 1:2,000) or tubulin (clone DM1A, Sigma-Aldrich/Merck, Darmstadt, Germany; 1:5,000). After washing, membranes were incubated for 1 h at RT with IRDye-conjugated secondary antibodies (goat anti-mouse IRDye 800CW, 1:10,000; or goat anti-mouse IRDye 680LT, 1:20,000; LI-COR Biosciences), washed again, and imaged using an Odyssey CLx Imager (LI-COR Biosciences). Band intensities were quantified using Image Studio Lite software (version 5.2; LI-COR Biosciences).

### Immune cell isolation and flow cytometry

Spleens were mechanically dissociated by passage through a 70-µm cell strainer, followed by red blood cell lysis using ammonium chloride–based lysis buffer (155 mM NH₄Cl, 10 mM KHCO₃, 0.1 mM EDTA) for 3 min at room temperature. Lysis was stopped by addition of FACS buffer (PBS containing 2% FCS and 2 mM EDTA), and cells were centrifuged and resuspended in FACS buffer. Splenocytes were used primarily for single-stain controls. Liver tissue was minced and digested in RPMI supplemented with 0.1% bovine serum albumin (BSA), 1% penicillin/streptomycin, and 0.2% collagenase type IV (Worthington Biochemical Corporation, Lakewood, NJ, USA) for 20 min at 37 °C. DNase I (0.9 mg/mL) was subsequently added, and digestion was continued for an additional 20 min. Digestion was stopped using HBSS containing 0.1% BSA and 2 mM EDTA. Cell suspensions were filtered through a 70-µm strainer, washed three times with PBS (centrifugation at 55 × g for 1 min at 4 °C), and subjected to density gradient centrifugation using 30% Nycodenz™ (Serumwerk Bernburg, Bernburg, Germany). Gradients were centrifuged at 1400 × g for 22 min at 4 °C with slow acceleration and no brake. Leukocytes were collected from the interphase, washed, resuspended in FACS buffer, and counted using a Neubauer hemocytometer. Heart tissue (20 mg) was minced and incubated in homogenization medium of RPMI (Gibco/Life Technologies, Carlsbad, California, USA) supplemented with 2% FCS, 1% penicillin/streptomycin, 30 mM HEPES, 1 mg/mL collagenase type II (Worthington Biochemical Corporation, Lakewood, NJ, USA), and 0.15 mg/mL DNase I (Sigma Aldrich, Burlington, Massachusetts, USA) under continuous shaking for 30 min at 37 °C. Enzymatic reaction was terminated by addition of 10 mM EDTA. Cell suspensions were passed through a 70-µm cell strainer, followed by red blood cell lysis as described above. Lysis was stopped by addition of FACS buffer (PBS containing 2% FCS and 2 mM EDTA), and cells were centrifuged and resuspended in FACS buffer. All cell suspensions were kept on ice until staining. Immune cells isolated from heart, spleen, and liver were incubated with Fc-blocking reagent (1:50) for 20 min at 4 °C, followed by staining with antibody cocktails for 20 min at 4 °C in the dark. Antibodies were obtained from BD Biosciences, BioLegend, and Life Technologies and are listed in Supplementary Table S1. Cells were washed and subsequently stained with Fixable Viability Dye (eFluor™ 780; 1:1000 dilution; eBioscience, San Diego, California, USA) for 30 min at 4 °C in the dark. Cells were then washed and fixed in 2% ROTI^TM^Histofix for 30 min at room temperature, followed by a final wash and resuspension in FACS buffer. For absolute quantification, 123count eBeads (Thermo Fisher Scientific, Waltham, MA, USA) were added to heart samples prior to acquisition. Flow cytometric acquisition was performed on a BD FACSymphony™ A3 analyzer, and data were analyzed using FlowJo software version 10.9.0. Detailed gating strategies are provided in Supplementary Fig. S7–S12.

### Statistics

**Fig. 1:**
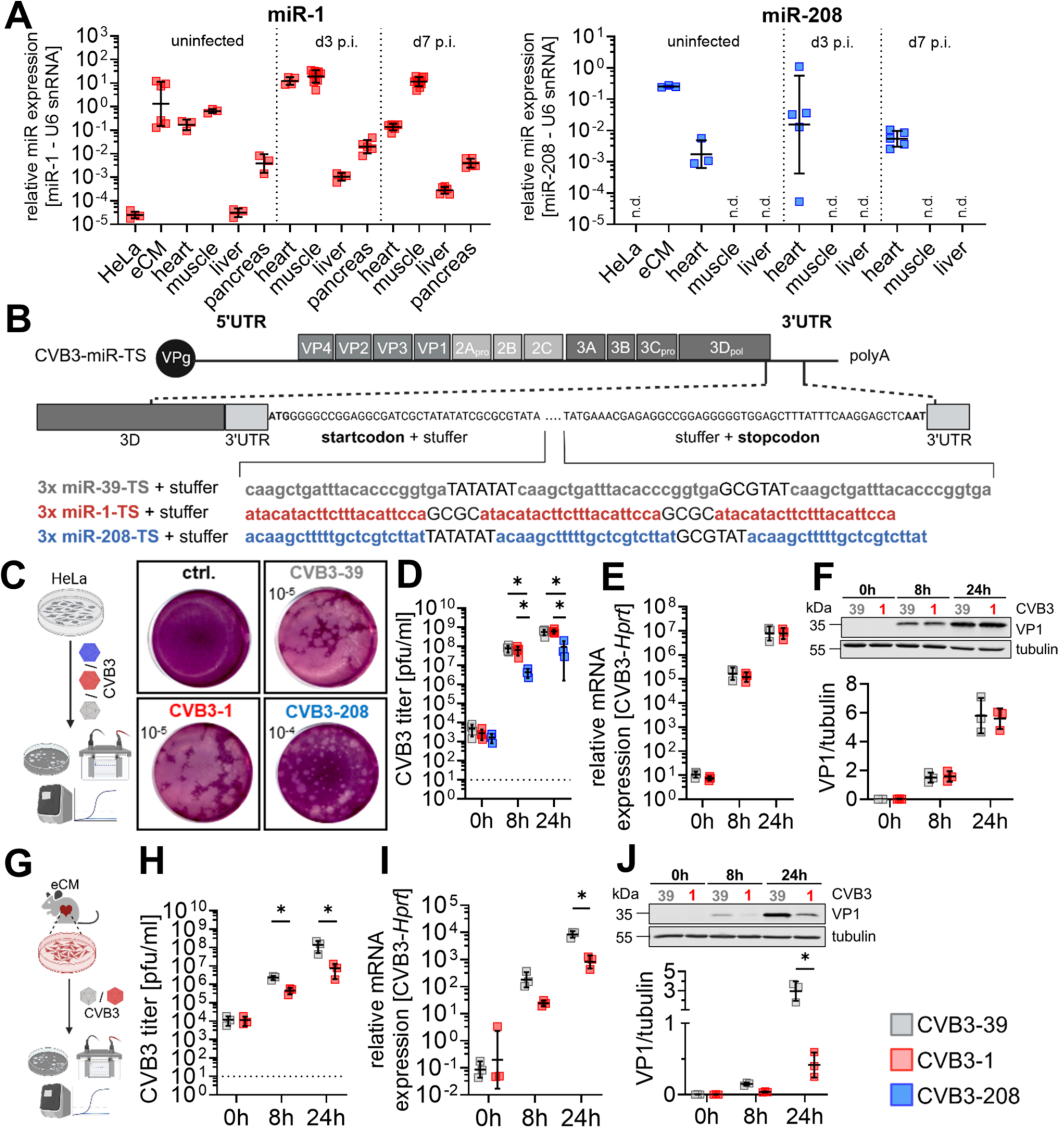
miR-1 targeting attenuates CVB3 replication in primary cardiomyocytes. **A** miR-1 (left panel, red squares) and miR-208 (right panel, blue squares) expression in uninfected cell lines and in uninfected and infected organs at days 3 and 7 post infection, normalized to U6 snRNA as an endogenous control (miR-1: HeLa *N* = 6, eCM *N* = 6, uninfected organs *N* = 3, d3 *N* = 5 (heart, m. gastrocnemius, m. quadriceps fem, liver, pancreas), d7 *N* = 6 (heart, m. gastrocnemius, m. quadriceps fem, liver, pancreas); miR-208: HeLa *N* = 3, eCM *N* = 3, uninfected organs *N* = 3, d3 *N* = 5 (heart, m. gastrocnemius, m. quadriceps fem, liver), d7 *N* = 5 (heart, m. gastrocnemius, m. quadriceps fem, liver)). If a specific miRNA was not detected, it is indicated as n.d. (= not determined). **B** Schematic representation of recombinant CVB3 constructs (adapted from [38]): CVB3-1 and CVB3-208, generated by insertion of miR-1 or miR-208 target sites (TS), respectively, into the 3′ untranslated region (UTR). In the same region, the control virus CVB3-39 contains the miR-39 TS, a non-mammalian microRNA target sequence [38]. **C** HeLa cells were infected with CVB3-39, CVB3-1 and CVB3-208 at an MOI of 1 and harvested at 0, 8 and 24 h post infection. Representative images of plaque sizes from the 8 h time point are shown. **D** Viral titers from cell lysates in **C** were determined by plaque assay (*N* = 3). (**E/F**) HeLa cells were infected with MOI 1 of CVB3-39 and CVB3-1 and harvested at 0, 8 and 24 h post infection (*N* = 3). **E** Viral genome expression was quantified by RT-qPCR. **F** VP1 protein expression was analyzed by Western blot using tubulin as a loading control. One representative blot and densitometric quantification of all replicates are shown **G** Schematic experimental design for in vitro infection of embryonal cardiomyocytes (eCM). Cells were infected with an MOI 5 of CVB3-39 or CVB3-1 and harvested at 0, 8, and 24 h post infection (*N* = 3). **H** Viral titers in eCM lysates were determined by plaque assay. **I** Viral genome expression was quantified by RT-qPCR. **J** VP1 protein expression was analyzed by Western blot using tubulin as a loading control. One representative blot and quantification of all replicates are shown. Data are mean ± SD. Outliers were detected using the ROUT (Q = 1%) method (A). Two-way ANOVA with Sidak's post hoc test was used for datasets with two independent factors (time and virus strain) (D-F, H-J). Statistical significance was defined as p ≤ 0.05 and is indicated by a single asterisk (*)

**Fig. 2:**
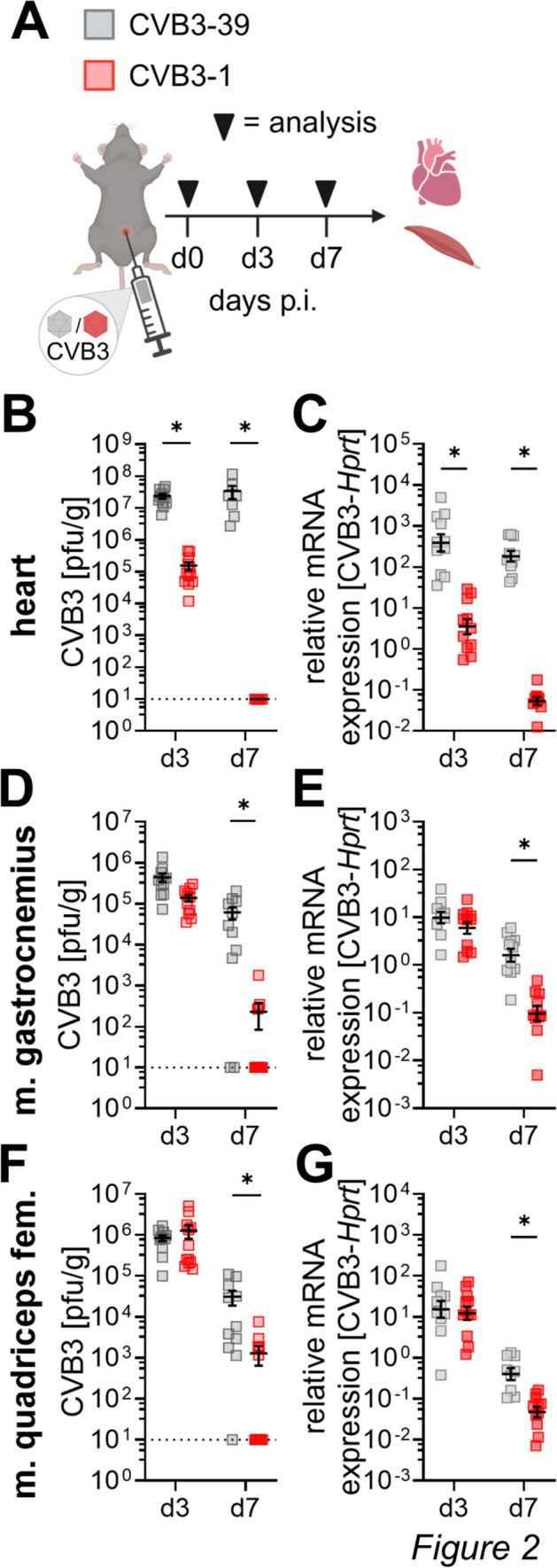
CVB3-1 variant demonstrates cardiac attenuation in vivo. **A** Experimental design. Six- to seven-week-old C57BL/6J mice were infected intraperitoneally with 10^5^ PFU of CVB3-39 or CVB3-1 and analyzed at day 3 (acute phase) and day 7 (subacute phase). Age-matched uninfected mice served as baseline controls (day 0). Group sizes: d0 (*N* = 8), d3 CVB3-39 (*N* = 12), d3 CVB3-1 (*N* = 12), d7 CVB3-39 (N = 12), d7 CVB3-1 (*N* = 13). Viral burden was quantified in the heart (**B, C**), musculus gastrocnemius (**D, E**), and musculus quadriceps femoris (**F, G**). Infectious viral titers were determined by plaque assay (**B, D, F**), and viral genome levels were quantified by RT-qPCR (**C, E, G**). Data are mean ± SD. Outliers were detected using the ROUT (Q = 1%) method (B-G). Two-way ANOVA with Sidak's post hoc test was used for datasets with two independent factors (time and virus strain) (B-G). Statistical significance was defined as *p* ≤ 0.05 and is indicated by a single asterisk (*)

**Fig. 3:**
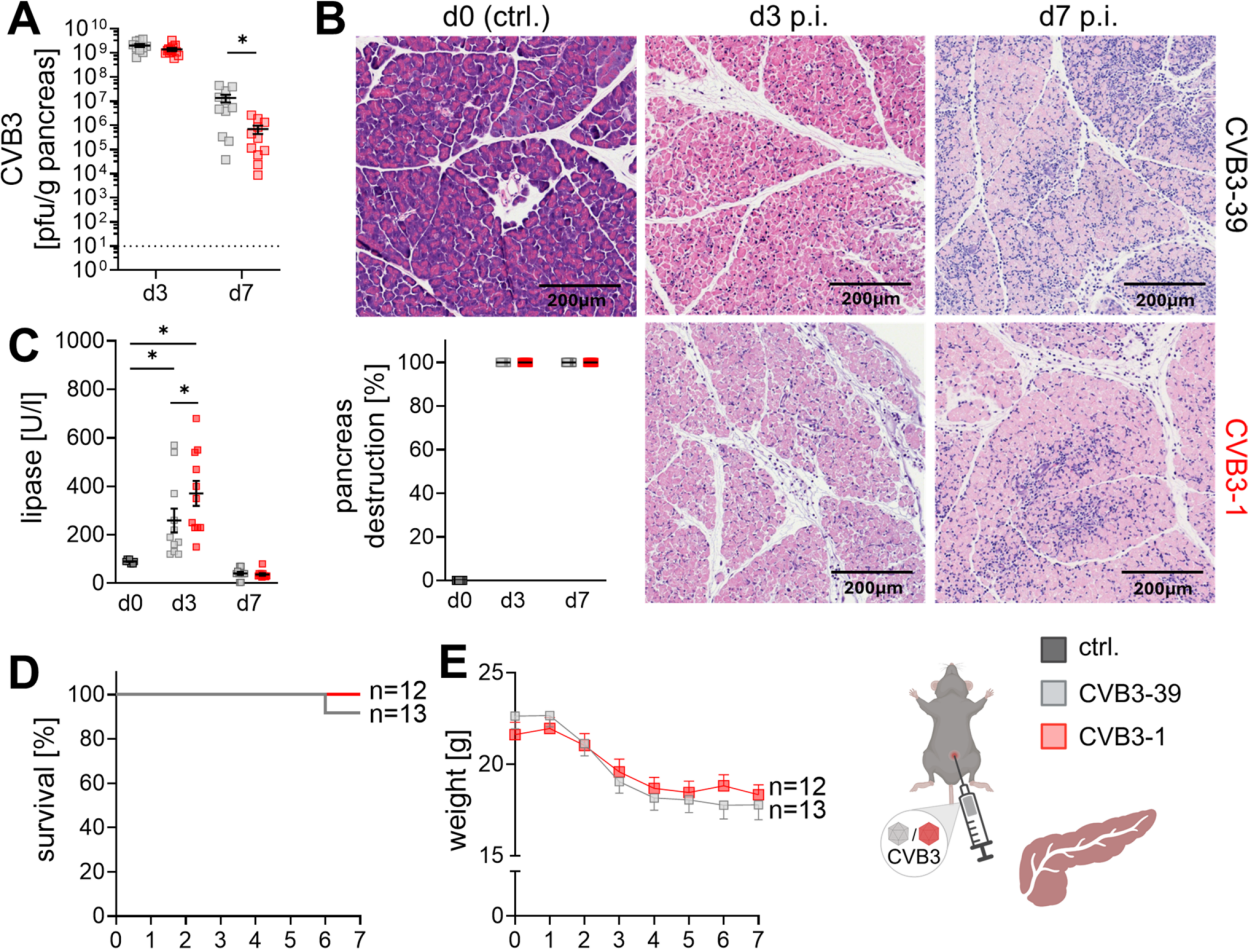
CVB3-1 infection does not alter pancreatic pathology. C57BL/6J were intraperitoneally infected with 10^5^ PFU of CVB3-39 or CVB3-1 and analyzed at day 3 (acute phase) and day 7 (subacute phase). Age-matched uninfected mice served as baseline controls (day 0). Group sizes: d0 (*N* = 8), d3 CVB3-39 (*N* = 12), d3 CVB3-1 (*N* = 12), d7 CVB3-39 (*N* = 12), d7 CVB3-1 (*N* = 13). Blood and pancreatic tissue were collected for analysis. **A** Pancreatic viral titers were determined by plaque assay; the limit of detection was 10^1^ PFU/g. **B** Representative hematoxylin and eosin (H&E)–stained pancreatic sections are shown for each time point. Scale bar, 200 µm. Pancreatic destruction was scored from 0 to 100%. **C** Serum lipase levels were measured from whole blood samples. **D** Kaplan–Meier survival curves. **E** Body weight change over the course of infection. Data are mean ± SD. Outliers were detected using the ROUT (Q = 1%) method (A/C). Two-way ANOVA with Sidak's post hoc test was used for datasets with two independent factors (time and virus strain) (A-C). One-way ANOVA with Dunnett's post hoc test compared post-infection time points to baseline (A-C). Survival curves were analyzed using log-rank (Mantel–Cox) test (D). Mixed effect model (REML) was used for repeated measurements (E). Statistical significance was defined as *p* ≤ 0.05 and is indicated by a single asterisk (*)

**Fig. 4:**
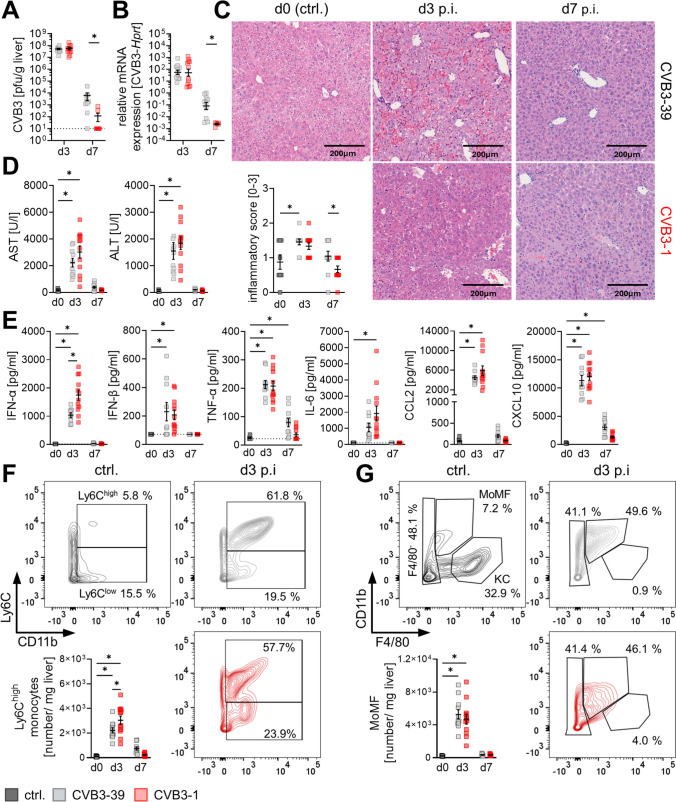
CVB3-1 induces acute hepatitis with preserved interferon responses. Six- to seven-week-old C57BL/6J mice were infected intraperitoneally with 10^5^ PFU of CVB3-39 or CVB3-1 and analyzed at day 3 (acute phase) and day 7 (subacute phase). Age-matched uninfected mice served as baseline controls (day 0). Group sizes were: d0 (*N* = 8), d3 CVB3-39 (*N* = 12), d3 CVB3-1 (*N* = 12), d7 CVB3-39 (*N* = 12), and d7 CVB3-1 (*N* = 13). **A** Hepatic viral burden was assessed by plaque assay (limit of detection: 10^1^ PFU/g) and **B** by RT-qPCR quantification of viral genomes. **C** Representative H&E–stained liver sections at the indicated time points. Scale bar, 200 µm. Liver inflammation and necrosis were scored according to the inflammatory scoring system described by [50]. **D** Serum aspartate aminotransferase (AST) and alanine aminotransferase (ALT) levels. **E** Serum interferon (IFN), cytokine, and chemokine levels measured using a LegendPlex assay. **F, G** Hepatic immune cell populations were analyzed by flow cytometry. Representative contour plots are shown for uninfected mice and at day 3 post infection for both viral strains. **F** CD45^+^lineage^−^CD11b^+^Ly6G^−^F4/80^−^CD11c^−^MHC-II^−^ Ly6C^high^ inflammatory and Ly6C^low^ patrolling monocytes and quantification of Ly6C^high^ cells per mg liver tissue. **G** CD45^+^lineage^−^CD11b^high^Ly6G^−^F4/80^intermediate^ monocyte-derived macrophages (MoMF) together with CD45^+^lineage^−^CD11b^intermediate^Ly6G^−^F4/80.^high^ Kupffer cells and quantification of MoMF per mg liver tissue. Data are mean ± SD. Outliers were detected using the ROUT (Q = 1%) method (A-B, D-F). Two-way ANOVA with Sidak's post hoc test was used for datasets with two independent factors (time and virus strain) (A-B/D-G). One-way ANOVA with Dunnett's post hoc test compared post-infection time points to baseline (D-G). Ordinal data were evaluated using a Mann–Whitney U test (C). Statistical significance was defined as *p* ≤ 0.05 and is indicated by a single asterisk (*)

**Fig. 5:**
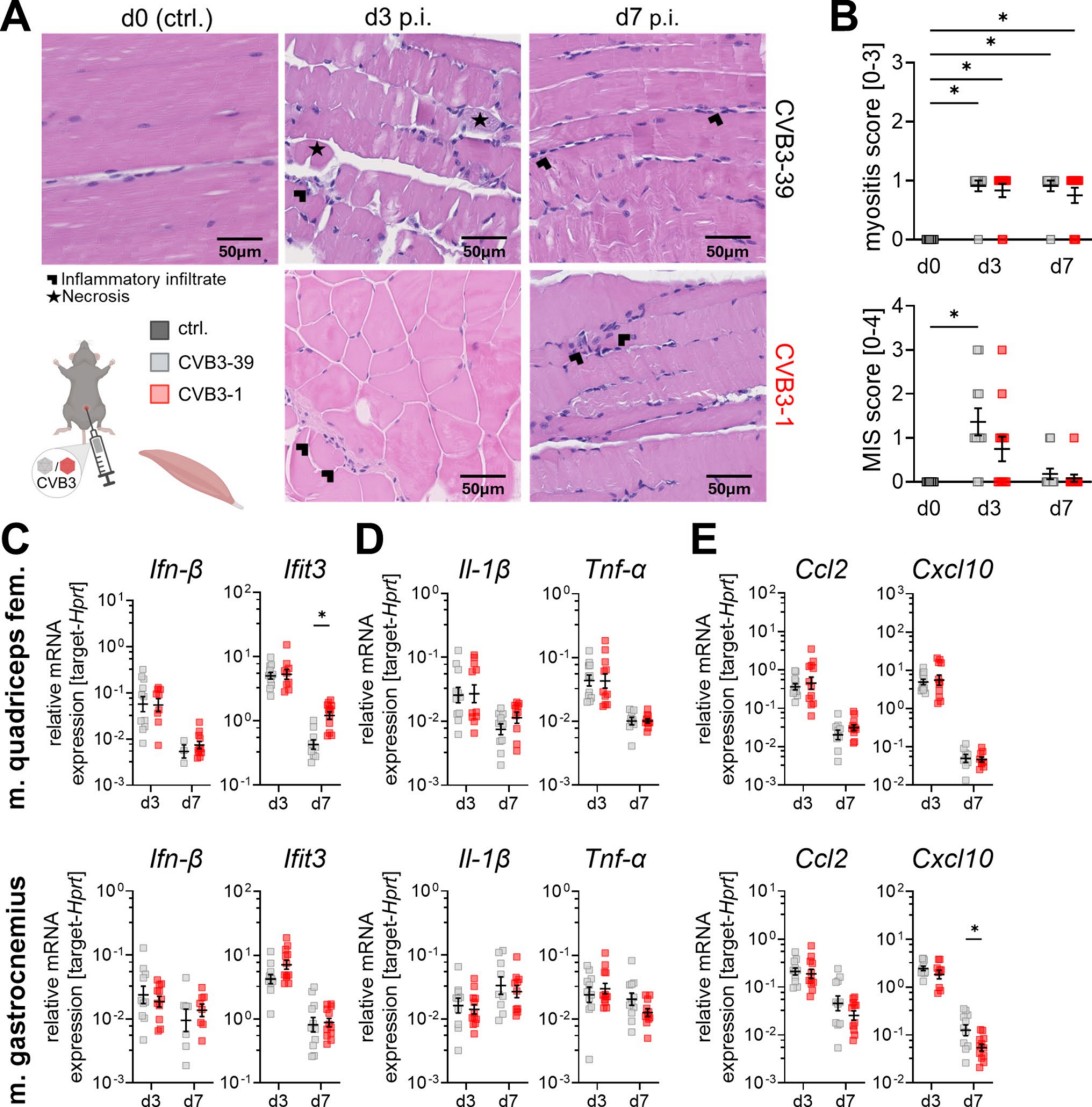
CVB3 infection induces transient and mild skeletal muscle pathology independent of strain. Six- to seven-week-old C57BL/6J mice were infected intraperitoneally with 10^5^ PFU/mouse of CVB3-39 or CVB3-1 and analyzed at day 3 (acute phase) and day 7 (subacute phase). Age-matched uninfected mice served as baseline controls (day 0). Musculus quadriceps femoris and musculus gastrocnemius were harvested for analysis (d0 *N* = 8; d3 CVB3-39 *N* = 12; d3 CVB3-1 *N* = 12; d7 CVB3-39 *N* = 12; d7 CVB3-1 *N* = 13). **A** Representative images from H&E-stained muscle sections at the indicated time points. Inflammatory infiltrates are indicated by arrows and necrotic areas by asterisks. Scale bar, 50 µm. **B** Histological quantification of myositis and muscle inflammatory score (MIS). **C-E** mRNA expression of interferon/interferon-stimulated genes (IFN/ISG) **C**, cytokines **D**, and chemokines **E** in muscle tissue measured by RT-qPCR. Data are mean ± SD. Outliers were detected using the ROUT (Q = 1%) method (C-E). Two-way ANOVA with Sidak's post hoc test was used for datasets with two independent factors (time and virus strain) (C-E). Ordinal data were evaluated using the Mann–Whitney U test (B). Statistical significance was defined as *p* ≤ 0.05 and is indicated by a single asterisk (*)

**Fig.6:**
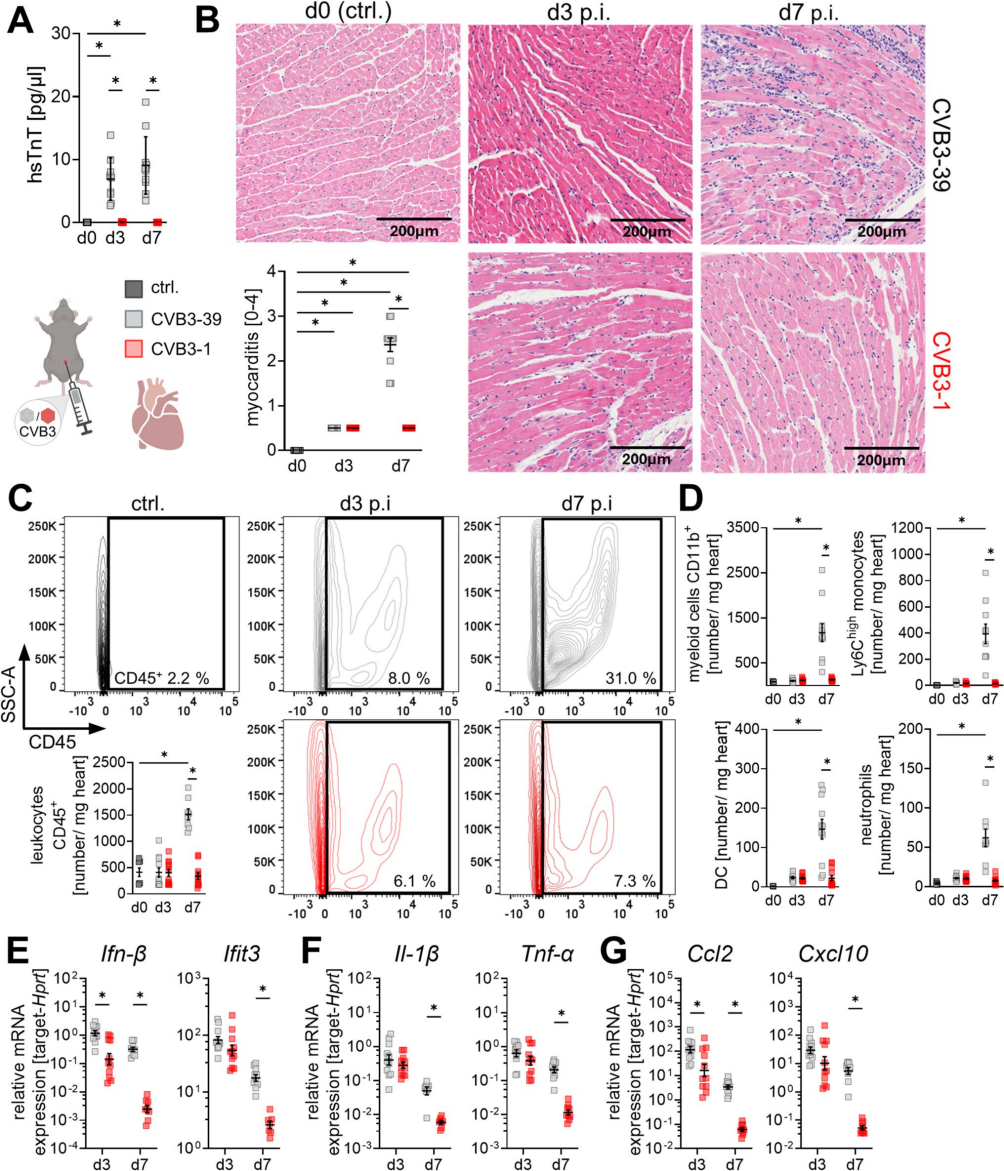
Myocarditis is abolished in CVB3-1 infection. Six- to seven-week-old C57BL/6J mice were infected intraperitoneally with 10^5^ PFU/mouse of CVB3-39 or CVB3-1 and analyzed at day 3 (acute phase) and day 7 (subacute phase). Age-matched uninfected mice served as baseline controls (day 0). Heart tissue was harvested for analysis (d0 *N* = 8; d3 CVB3-39 *N* = 12; d3 CVB3-1 *N* = 12; d7 CVB3-39 *N* = 12; d7 CVB3-1 *N* = 13). **A** Serum troponin T levels were measured using an electrochemiluminescence immunoassay (ECLIA). **B** Representative images from H&E-stained heart sections at the indicated time points. Myocarditis was scored histologically. Scale bar, 200 µm. **C, D** Cardiac immune cell populations were analyzed by flow cytometry. **C** Representative contour plots of CD45^+^ leukocytes for both viral strains with corresponding quantification. **D** Quantification of CD45⁺CD11b⁺ myeloid cells, CD45^+^CD11b^+^CD3^−^NK1.1^−^B220^−^Ly6G^−^CD11c^−^F4/80^−^Ly6C^high^ inflammatory monocytes, CD45^+^CD11b^+^CD3^−^NK1.1^−^B220^−^Ly6G^−^CD11c^+^F4/80^−^ dendritic cells (DC) and CD45^+^CD11b^+^CD3^−^NK1.1^−^B220^−^Ly6G^+^ neutrophils. **E–G** mRNA expression of interferon/interferon-stimulated genes (IFN/ISG) **E**, cytokines **F**, and chemokines **G** in cardiac tissue measured by RT-qPCR. Data are mean ± SD. Outliers were detected using the ROUT (Q = 1%) method (A/C-F). Two-way ANOVA with Sidak's post hoc test was used for datasets with two independent factors (time and virus strain) (A/C-G). One-way ANOVA with Dunnett's post hoc test compared post-infection time points to baseline (A/C-D). Ordinal data were evaluated using a Mann–Whitney U test (B). Statistical significance was defined as p ≤ 0.05 and is indicated by a single asterisk (*)

**Fig.7:**
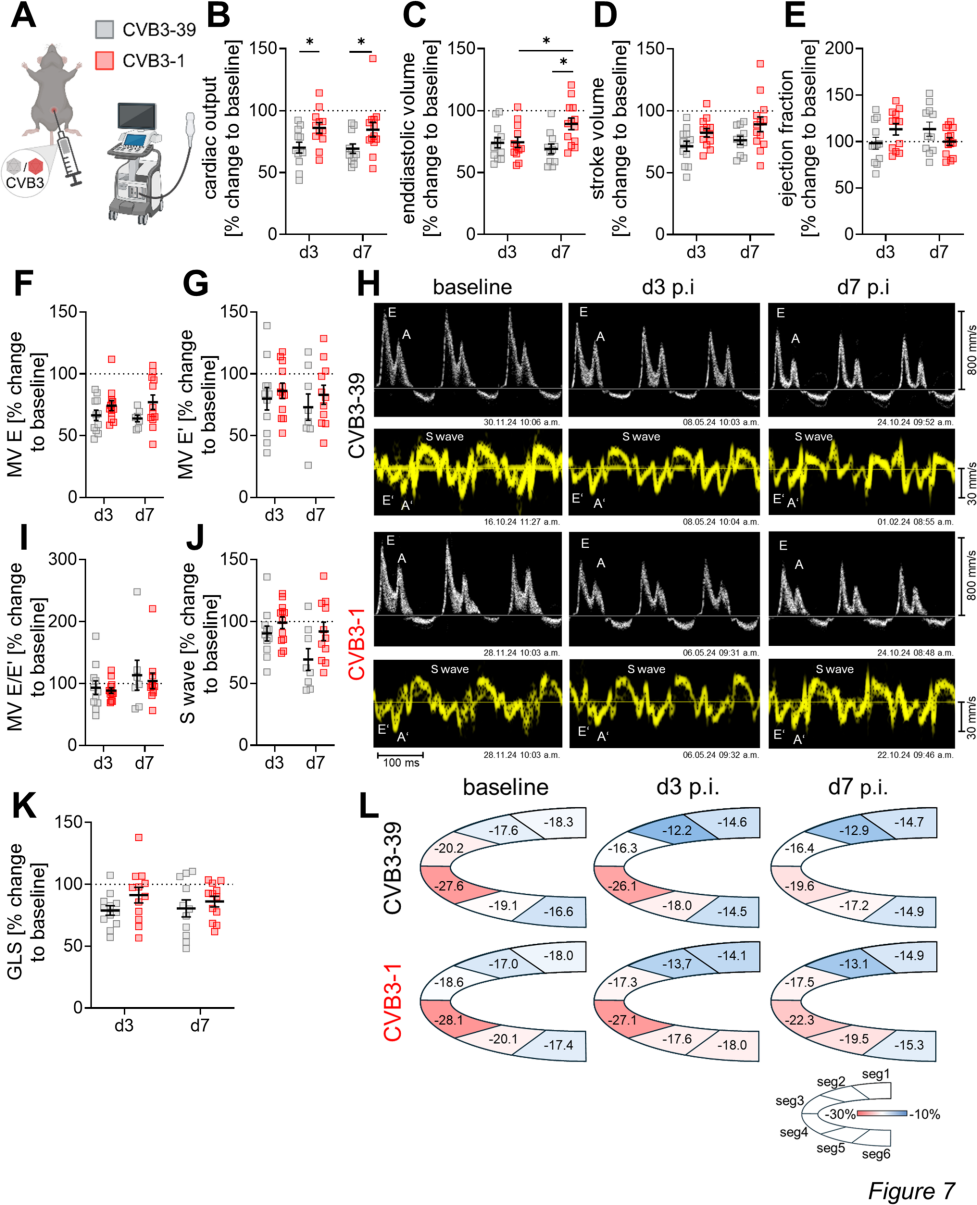
Echocardiographic analysis reveals early and persistent cardiac dysfunction independent of myocarditis. **A** Experimental design. C57BL/6J mice were infected intraperitoneally with 10^5^ PFU/mouse of CVB3-39 or CVB3-1. Transthoracic echocardiography was performed one day prior to infection (baseline) and at day 3 (acute phase) and day 7 (subacute phase) post infection immediately before sacrifice. Group sizes were as follows: baseline for d3 comparison: CVB3-39 *N* = 12, CVB3-1 *N* = 12, d3: CVB3-39 *N* = 12 and CVB3-1 *N* = 12; baseline for d7 comparison: CVB3-39 *N* = 11, CVB3-1 *N* = 13, d7: CVB3-39 *N* = 11, and CVB3-1 *N* = 13. Left ventricular systolic function and volumes were assessed as follows: cardiac output **B**, left ventricular end-diastolic volume (LVEDV) (**C**), stroke volume (SV) **D**, and ejection fraction (EF) **E**. **F–J** Doppler measurements of diastolic and systolic function. Mitral valve E-wave velocity (MV E) **F** and early diastolic myocardial velocity (E′) **G** are shown. **H** Representative pulsed-wave Doppler images of transmitral inflow (E and A waves, white profiles) and septal tissue Doppler imaging (E′ and S′ velocities, yellow profiles) at the indicated time points. **I** Left ventricular E/E′ ratio. **J** Tissue Doppler S′ wave velocity. **K-L** Global longitudinal strain (GLS) analysis. **K** Quantification of GLS. **L** Segmental longitudinal strain values (%) averaged across six left ventricular segments (seg1: anterior base; seg2: anterior mid; seg3: anterior apex; seg4: posterior apex; seg5: posterior mid; seg6: posterior base). Echocardiographic parameters were normalized to the individual baseline values of each animal and are presented as percentage change from baseline (B-G/I-K). Data are mean ± SD. Normalized data were used for statistical comparisons between virus groups at individual time points and for comparisons of the same virus strain across different days post-infection, applying unpaired t-tests or Mann–Whitney U tests when normality was not met (B-G/I-K). Statistical significance was defined as *p* ≤ 0.05 and is indicated by a single asterisk (*)

Statistical analyses were performed using GraphPad Prism version 10.5.0 for Windows (GraphPad Software, La Jolla, CA, USA). For data shown in Figs. 1A, 2B-G, 3A,C, 4A-B, D-G, 5C-E, 6A,C-F and Supplementary Fig. S1A-E; S2A-D; S3A-F; S4A-D, outliers were identified using the ROUT method (Q = 1%) and excluded from analysis. Data are presented as individual values with mean ± SD unless stated otherwise. Echocardiographic measurements were normalized to the individual baseline values of each animal and expressed as percentage change from baseline. Normality of data distribution was assessed using the D’Agostino–Pearson test. Viral titers are displayed on a log10-scaled y-axis and were log10-transformed prior to statistical analysis. Relative gene expression was calculated using the 2^−ΔCt^ method and log10-transformed for statistical testing and visualization. For echocardiographic analyses, normalized data were used for statistical comparisons between virus groups at individual time points and for comparisons of the same virus strain across different days post-infection, applying unpaired t-tests or Mann–Whitney U tests when normality was not met (Fig. 7B-G, I-K**; **Table 3). Changes in echocardiographic parameters within the same animal over time were analyzed using paired t-tests on the original (non-normalized) data (Table 1, 2).

**Table 1:** Longitudinal echocardiographic analysis in CVB3-1–infected mice

CVB3-1	baseline	day 3	Baseline vs day 3	baseline	day7	Baseline vs day 7
LV Performance						
Heart rate [bpm]	403.5 ± 21.3	421.2 ± 30.9	ns	421.6 ± 42.2	401.1 ± 38.7	ns
LVEDV [µl]	54.0 ± 9.0	39.7 ± 7.9	*	50.6 ± 7.6	44.5 ± 7.8	*
LVESV [µl]	26.7 ± 7.7	17.4 ± 6.4	*	24.4 ± 5.6	21.5 ± 5.0	ns
Stroke volume [µl]	27.3 ± 4.1	22.3 ± 3.5	*	26.1 ± 3.0	23.0 ± 4.8	ns
Cardiac output [ml/min]	11.0 ± 1.5	9.4 ± 1.7	*	11.0 ± 1.3	9.2 ± 2.2	*
EF [%]	51.2 ± 7.5	57.0 ± 9.5	ns	52.2 ± 5.4	51.8 ± 6.4	ns
M-Mode						
LVAWd [mm]	0.8 ± 0.2	0.9 ± 0.2	*	0.8 ± 0.1	0.9 ± 0.1	ns
LVPWd [mm]	0.7 ± 0.1	0.8 ± 0.1	*	0.7 ± 0.0	0.8 ± 0.2	*
LVIDd [mm]	3.9 ± 0.3	3.4 ± 0.2	*	3.8 ± 0.2	3.4 ± 0.2	*
LV Mass [mg]	108.4 ± 21.8	96.1 ± 18.8	ns	97.9 ± 12.5	94.9 ± 23.0	ns
PW-Doppler						
MV E [mm/s]	824.8 ± 78.8	609.5 ± 108.5	*	780.9 ± 131.2	587.0 ± 121.3	*
MV A [mm/s]	550.6 ± 110.2	431.3 ± 77.2	*	516.9 ± 6.2	419.2 ± 109.6	ns
MV E/A	1.5 ± 0.3	1.4 ± 0.2	ns	1.5 ± 0.2	1.5 ± 0.4	ns
IVRT [ms]	14.3 ± 1.6	14.3 ± 2.1	ns	12.7 ± 2.5	14.9 ± 3.1	*
IVCT [ms]	12.1 ± 3.0	12.9 ± 2.4	ns	13.0 ± 3.1	15.4 ± 5.8	ns
AET [ms]	49.3 ± 3.0	44.7 ± 5.1	*	51.1 ± 5.2	46.8 ± 5.5	*
Tissue-Doppler						
MV E' [mm/s]	−21.8 ± 5.6	−18.5 ± 5.9	ns	−19.3 ± 3.5	−16.1 ± 6.0	ns
MV A' [mm/s]	−30.5 ± 5.0	−22.5 ± 3.7	*	−26.5 ± 4.2	−20.4 ± 6.4	*
MV E'/A'	0.8 ± 0.2	0.8 ± 0.3	ns	0.7 ± 0.1	0.9 ± 0.8	ns
MV E/E'	−40.5 ± 12.4	−35.1 ± 8.8	*	−41.1 ± 7.3	−41.8 ± 14.2	ns
S' [mm/s]	27.0 ± 2.7	26.6 ± 4.2	ns	25.0 ± 3.0	22.6 ± 4.8	ns
Strain analysis						
GLS [%]	−19.7 ± 2.1	−17.7 ± 3.1	ns	−19.3 ± 2.0	−16.6 ± 3.1	*

C57BL/6J mice were infected intraperitoneally with 10^5^ PFU/mouse of CVB3-1. Transthoracic echocardiography was performed one day prior to infection (baseline) and at day 3 (acute phase) or day 7 (subacute phase) post infection immediately before sacrifice. Group sizes were as follows: baseline for d3 comparison (N = 12) with corresponding d3 measurements (N = 12); baseline for d7 comparison (N = 13) with corresponding d7 measurements (N = 13). Data are mean ± SD. Statistical analysis was performed using paired t-tests comparing matched measurements between baseline and day 3 or baseline and day 7. Statistical significance was defined as p ≤ 0.05 and is indicated by a single asterisk (*)

**Table 2:** Longitudinal echocardiographic analysis in CVB3-39–infected mice

CVB3-39	baseline	day 3	baseline vs day 3	baseline	day7	baseline vs day 7
LV Performance						
Heart rate [bpm]	452.4 ± 36.1	442.7 ± 33.7	ns	403.9 ± 18.8	415.6 ± 104.2	ns
LVEDV [µl]	49.4 ± 4.7	36.5 ± 7.5	*	52.4 ± 5.9	36.3 ± 8..1	*
LVESV [µl]	24.1 ± 3.6	18.7 ± 7.0	*	28.8 ± 4.9	18.2 ± 6.0	*
Stroke volume [µl]	25.3 ± 3.3	17.7 ± 2.5	*	23.7 ± 2.5	18.1 ± 3.5	*
Cardiac output [ml/min]	11.4 ± 1.7	7.9 ± 1.4	*	9.5 ± 0.9	6.6 ± 1.2	*
EF [%]	51.2 ± 5.3	49.9 ± 9.7	ns	45.4 ± 4.3	50.7 ± 8.1	ns
M-Mode						
LVAWd [mm]	0.9 ± 0.1	1.0 ± 0.1	ns	0.7 ± 0.0	1.0 ± 0.2	*
LVPWd [mm]	0.7 ± 0.1	0.7 ± 0.1	ns	0.7 ± 0.1	0.9 ± 0.1	*
LVIDd [mm]	3.8 ± 0.1	3.5 ± 0.3	*	3.8 ± 0.2	3.2 ± 0.4	*
LV Mass [mg]	105.4 ± 17.1	103.4 ± 21.7	ns	93.3 ± 10.2	99.9 ± 19.0	ns
PW-Doppler						
MV E [mm/s]	819.0 ± 132.9	534.4 ± 86.7	*	757.0 ± 115.8	477.4 ± 30.9	*
MV A [mm/s]	530.0 ± 82.8	415.2 ± 59.4	*	515.3 ± 111.2	300.1 ± 68.4	*
MV E/A	1.5 ± 0.1	1.3 ± 0.1	*	1.5 ± 0.3	1.7 ± 0.4	ns
IVRT [ms]	14.2 ± 1.1	14.7 ± 1.2	ns	13.3 ± 1.5	16.4 ± 2.3	*
IVCT [ms]	12.8 ± 2.2	13.4 ± 2.2	ns	13.3 ± 3.6	13.1 ± 1.7	ns
AET [ms]	48.4 ± 4.7	48.1 ± 7.2	ns	48.6 ± 3.8	47.4 ± 2.9	ns
Tissue-Doppler						
MV E' [mm/s]	−18.9 ± 6.2	−14.1 ± 4.7	*	−19.9 ± 4.3	−13.6 ± 3.6	*
MV A' [mm/s]	−31.4 ± 7.8	−21.3 ± 3.2	*	−27.9 ± 5.1	−15.9 ± 5.6	*
MV E'/A'	0.6 ± 0.3	0.7 ± 0.2	ns	0.7 ± 0.1	1.1 ± 0.8	ns
MV E/E'	−49.2 ± 23.0	−41.4 ± 13.7	ns	−38.5 ± 8.8	−40.7 ± 17.9	ns
S' [mm/s]	24.6 ± 3.4	22.3 ± 5.6	ns	25.9 ± 2.3	17.7 ± 5.9	*
Strain analysis						
GLS [%]	−20.4 ± 1.2	−16.0 ± 2.6	*	−18.6 ± 2.3	−15.0 ± 4.7	*

C57BL/6J mice were infected intraperitoneally with 10^5^ PFU/mouse of CVB3-39. Transthoracic echocardiography was performed one day prior to infection (baseline) and at day 3 (acute phase) or day 7 (subacute phase) post infection immediately before sacrifice. Group sizes were as follows: baseline for d3 comparison (N = 12) with corresponding d3 measurements (N = 12); baseline for d7 comparison (N = 11) with corresponding d7 measurements (N = 11). Data are mean ± SD. Statistical analysis was performed using paired t-tests comparing matched measurements between baseline and day 3 or baseline and day 7. Statistical significance was defined as p ≤ 0.05 and is indicated by a single asterisk (*)

Datasets comprising two independent factors (time and virus strain) were analyzed using two-way ANOVA (Figs. 1D-F, H-J, 2B-G, 3A, 4A,B 5C-E, 6E-G; Supplementary Fig.S2B-D). Where repeated measurements within the same animals were available, a mixed effect model (REML) was applied (Fig. 3E). Post hoc comparisons were performed using Sidak’s multiple-comparison test. Comparisons of post-infection time points to baseline were conducted using one-way ANOVA followed by Dunnett’s multiple-comparison test (Figs. 3B,C, 4D-G, 6A,C-D, Supplementary Fig. S1A-E; S2A; S3A-F; S4A-D). Direct comparisons among infected animals were done by ordinary two-way ANOVA (factors: virus and time), followed by Sidak’s multiple comparison test (Figs. 3B,C, 4D-G, 6A,C-D; Supplementary Fig. S1A-E; S2A; S3A-F; S4A-D). Survival curves were analyzed using the log-rank (Mantel–Cox) test (Fig. 3D), and ordinal or non-normally distributed data were evaluated using the Mann–Whitney U test (Figs. 4C; 5B; 6B). Statistical significance was defined as *p* ≤ 0.05.

## Results

### Establishment of microRNA-targeted CVB3 variants for cardiac attenuation

To identify candidate microRNAs for cardiac detargeting, we first screened publicly available datasets from the Mouse Expression Atlas to define microRNAs with enriched expression in cardiac tissue [19]. Due to their reported muscle-associated expression patterns, miR-1 and miR-208 emerged as leading candidates. We then experimentally assessed whether their expression profiles were altered by CVB3 infection in mice by quantifying miR-1 and miR-208 levels in heart, muscle, liver and pancreatic tissue at day 3 and day 7 after infection. These data were complemented by expression analyses in embryonic cardiomyocytes and HeLa cells (Fig. 1A). miR-1 displayed high and consistent expression in cardiac and skeletal muscle, with negligible expression in liver, pancreas and HeLa cells. Its cardiac expression increased during acute infection (day 3) and returned toward baseline levels by day 7. miR-208 was expressed at lower absolute levels but showed pronounced cardiac specificity. Upon infection, miR-208 regulation was more heterogeneous, with partially inverse regulation compared to miR-1 across individual animals, while overall tissue selectivity was preserved. The complementary features of miR-1 and miR-208 in CVB3 infection motivated the parallel evaluation of both microRNAs as potential tools for cardiac detargeting in subsequent experiments.

To generate the cardiac-detargeted CVB3 variants, three tandem target sites for miR-1 or miR-208 were inserted into the 3′ untranslated region (3′UTR) of the CVB3 genome, yielding CVB3-miR-1-TS (CVB3-1) and CVB3-miR-208-TS (CVB3-208) (Fig. 1B). All three target site cassettes were positioned in the same region of the viral 3′UTR downstream of the coding sequence, ensuring comparability between constructs and minimizing confounding effects related to insertion site or genome architecture (Fig. 1B) [14]. A construct carrying three tandem target sites for miR-39, a non-mammalian microRNA derived from *Caenorhabditis elegans*, served as a replication-competent control virus (CVB3-39) [38]. Replication competence of the engineered CVB3 variants was first assessed in HeLa cells, which lack detectable expression of both miR-1 and miR-208 (Fig. 1A). Under these non-target conditions, CVB3-208 formed markedly smaller plaques and exhibited substantially reduced viral titers compared with both CVB3-39 and CVB3-1, indicating an intrinsic replication defect (Fig. 1C-D). CVB3-1 displayed cytopathic effects and replication kinetics indistinguishable from CVB3-39 (Fig. 1C-D). Accordingly, CVB3-208 was excluded from further analyses, and CVB3-1 was selected for all subsequent experiments. Viral RNA levels and expression of the capsid protein VP1 were comparable between CVB3-1 and CVB3-39 at both genome (Fig. 1E) and protein levels (Fig. 1F), confirming similar viral replication properties of CVB3-1 and CVB3-39 in HeLa cells.

To investigate cardiomyocyte-specific detargeting, primary murine embryonal cardiomyocytes, shown to express miR-1 (Fig. 1A), were infected with either CVB3-1 or CVB3-39 (Fig. 1G). In comparison to CVB3-39, CVB3-1 infection in cardiomyocytes resulted in significantly reduced infectious titers (Fig. 1H), viral RNA levels (Fig. 1I), and VP1 protein expression (Fig. 1J). Virus attenuation was already evident 8 h post infection and became more pronounced at 24 h (Fig. 1H-J). Thus, CVB3-1 selectively suppresses viral replication in cardiomyocytes while maintaining full replication competence in HeLa cells. Collectively, these data establish miR-1-mediated detargeting as a robust and specific strategy to uncouple cardiac from systemic CVB3 replication.

### Selective in vivo attenuation of cardiac CVB3 replication by miR-1 detargeting

To determine whether miR-1-mediated detargeting confers selective attenuation of CVB3 replication in vivo, C57BL/6J mice were infected i.p. with 10^5^ PFU of CVB3-1 or the control virus CVB3-39, and viral loads were quantified during the acute (day 3) and subacute (day 7) phases of infection (Fig. 2A). In the heart, CVB3-1 infection resulted in a marked reduction of viral titers already at day 3 (≈3-log decrease compared with control virus) (Fig. 2B). This divergence became even more pronounced at day 7, when cardiac titers in CVB3-39-infected mice remained high and largely unchanged compared with day 3, indicating sustained myocardial replication, whereas CVB3-1 titers were reduced by ≈6–7 logs, approaching the limit of detection (Fig. 2B). Quantification of viral genome copy numbers closely paralleled these findings: cardiac CVB3 genomes were significantly reduced in CVB3-1-infected mice at both day 3 and day 7, with near-complete clearance at the subacute stage, confirming effective suppression of myocardial replication at both the infectious and genomic levels (Fig. 2C).

Since miR-1 is expressed at similarly high levels in skeletal muscle (Fig. 1A), we quantified infectious virus in both the gastrocnemius and quadriceps femoris muscles. Virus titer in skeletal muscle was substantially lower than in the heart. In the gastrocnemius muscle, titers declined from day 3 to day 7 in both virus groups, with no separation at day 3 and only modest differences at day 7 (Fig. 2D). A similar pattern was observed in the quadriceps femoris muscle, where viral loads were already low during the acute phase and decreased further by day 7, again with differences between CVB3-1 and CVB3-39 restricted to day 7 (Fig. 2F). Viral genome quantification yielded congruent results: genome copy numbers in both skeletal muscles decreased from day 3 to day 7 and were significantly lower in CVB3-1-infected mice at day 7, consistent with the observed titer reductions (Fig. 2E,G). Altogether, these data demonstrate that miR-1 detargeting selectively abrogates sustained cardiac replication at both the infectious and genomic level, while skeletal muscle involvement remains limited and restricted to the subacute phase.

### miR-1 detargeting does not alter pancreatic replication or systemic disease severity

Because the exocrine pancreas represents the primary replication hub of CVB3 following intraperitoneal infection and is indispensable for establishing viremia and subsequent cardiac seeding [38], we examined whether miR-1 detargeting exerts off-target attenuation in this critical organ. Quantification of infectious virus revealed high pancreatic titers at day 3 post infection that were indistinguishable between CVB3-1- and control-virus-infected mice, demonstrating intact early replication despite insertion of miR-1 target sites (Fig. 3A). By day 7, pancreatic viral loads declined markedly in both groups, consistent with progressive tissue destruction and loss of permissive cells, with a modest reduction in CVB3-1-infected animals (Fig. 3A). These late differences occurred in the context of comparable early replication. Histological examination corroborated these findings: H&E staining demonstrated fulminant exocrine pancreatic destruction with dense inflammatory infiltration already at day 3 and persisting at day 7 in both groups (Fig. 3B). Quantitative lesion scoring confirmed near-complete pancreatic damage by day 3 irrespective of the viral strain (Fig. 3B). In line with the histopathology, serum lipase activity was markedly elevated during the acute phase and declined toward day 7 as functional pancreatic tissue was progressively lost (Fig. 3C). Altogether, miR-1 detargeting largely preserved the severity of virus-induced pancreatitis.

Consistently, overall systemic disease severity was comparable between groups. Survival was preserved throughout the observation period (Fig. 3D), and both CVB3-1 and control virus induced a similar degree of morbidity, reflected by a progressive reduction in absolute body weight by day 7 (Fig. 3E). Consistent with this shared systemic phenotype, organ-weight readouts, including depletion of inguinal white adipose tissue, liver mass changes, and skeletal and heart muscle mass trajectories – were largely indistinguishable between groups (Supplementary Fig. S1**A-E**). Together, these data indicate that miR-1 detargeting largely preserves pancreatic replication and key features of systemic disease, while enabling experimental dissection of cardiac-specific consequences of viral replication.

### miR-1 detargeting does not affect viral hepatitis during acute CVB3 infection

Beyond the pancreas, the liver represents a central target organ of systemic CVB3 infection. It serves as a key inducer of type I interferon responses, and a metabolic hub that undergoes rapid infection-induced metabolic reprogramming, thereby contributing substantially to systemic pathophysiology [24, 28]. We therefore next examined whether miR-1 detargeting exerts off-target effects on hepatic viral replication, immune activation, or tissue injury. Quantification of hepatic viral burden revealed high infectious titers (Fig. 4A) and viral genome copy numbers (Fig. 4B) at day 3 post infection that were indistinguishable between CVB3-1 and control virus. By day 7, viral titers and genomes declined markedly in both groups, with a more pronounced reduction in CVB3-1-infected mice (Fig. 4A,B). Histopathological analysis confirmed a severe but transient acute hepatitis (Fig. 4C). At day 3, H&E staining demonstrated multifocal inflammatory infiltrates and hepatocellular injury in both groups, which largely resolved by day 7. Quantitative scoring revealed no differences in the extent of acute liver pathology between CVB3-1 and control virus (Fig. 4C). Consistent with histological evidence of hepatocellular injury, serum transaminases (AST, ALT) (Fig. 4D) and LDH (Supplementary Fig. S2A) were markedly elevated at day 3 and declined toward near-baseline levels by day 7, whereas albumin levels were transiently reduced during acute infection (Supplementary Fig. S2A). These biochemical changes were comparable between virus groups. Transcriptional profiling showed a robust antiviral and inflammatory response during the acute phase (Supplementary Fig. S2B-D). Hepatic expression of interferon-β (*Ifn-β*) and the interferon-stimulated gene *Ifit3 –* selected as a representative ISG – was strongly induced at day 3 and declined by day 7. This antiviral signature was accompanied by marked upregulation of pro-inflammatory cytokines (*Il-1β*, *Tnf-α*) and chemokines (*Ccl2*, *Cxcl10*), which similarly peaked during the acute phase and normalized thereafter (Supplementary Fig. S2B-D). None of these transcriptional responses differed between virus groups except for minor variations for *Ifit3* in the acute and *Tnf-α* in the sub-acute phase.

To complement the tissue-level transcriptional analyses and to capture the systemic inflammatory milieu during acute infection, we additionally quantified circulating interferons, cytokines, and chemokines in serum using multiplex bead-based assays (Fig. 4E). Consistent with the hepatic interferon, cytokine and chemokine signatures (Supplementary Fig. S2B-D), serum levels of IFN-α, IFN-β, TNF-α, IL-6, and the chemokine CCL2 and CXCL10 were markedly elevated at day 3 post infection and declined toward baseline by day 7 (Fig. 4E). Cytokine profiles were broadly similar between CVB3-1- and control-virus-infected mice, although differences were observed for selected cytokines at specific time points. IFN-α levels were higher during the acute phase of CVB3-1 infection, while IFN-β levels did not differ between the viral strains (Fig. 4E). These data indicate a robust but transient systemic inflammatory response during acute CVB3 infection, likely driven by major target organs such as the pancreas and liver, and further confirm that miR-1 detargeting largely retains systemic interferon or cytokine responses.

To further characterize the hepatic immune response at the cellular level, we next analyzed liver-infiltrating immune cells by flow cytometry (Supplementary Fig. S7–S10 for gating strategy). Acute infection was characterized by a pronounced influx of myeloid cells at day 3, dominated by Ly6C^high^ monocytes (Fig. 4F) and monocyte-derived macrophages (MoMF) (Fig. 4G), while resident Kupffer cells were transiently reduced (Supplementary Fig. S3C). By day 7, immune cell numbers returned toward baseline (Supplementary Fig. S3A-F). Lymphoid populations, including B, T and NK cells, were present at low frequencies relative to the myeloid compartment (Supplementary Fig. S3D-F). Infection with CVB3-1 lead to slightly higher amounts of Ly6C^high^ monocytes and T cells in the acute phase (Fig. 4F, Supplementary Fig. S3E). Altogether, CVB3 infection induced a fulminant but self-limiting acute hepatitis. Importantly, miR-1 detargeting did not substantially alter the severity of acute liver pathology, suggesting that its biological effects are largely restricted to the myocardium, while systemic disease manifestations remain broadly comparable between groups.

### Skeletal muscle pathology is mild and unaffected by miR-1 detargeting

To ensure that miR-1-mediated viral detargeting does not introduce off-target effects in other miR-1-expressing tissues, we next assessed skeletal muscle pathology during CVB3 infection. As shown above, miR-1 detargeting did not alter skeletal muscle viral loads during the acute phase, and minor differences were observed at day 7 (Fig. 2D-G). Histological analysis of m. gastrocnemius and m. quadriceps femoris revealed a mild myositis at day 3, characterized by limited inflammatory infiltrates and occasional myofiber damage, which largely resolved by day 7 (Fig. 5A). Quantitative assessment confirmed low-grade muscle involvement, without differences between CVB3-1- and control-virus-infected mice (Fig. 5B). Transcriptional profiling demonstrated a modest induction of innate immune responses in skeletal muscle. *Ifn-β* and *Ifit3* (Fig. 5C), as well as the proinflammatory cytokines *Il-1β* and *Tnf-α* (Fig. 5D) and chemokines *Ccl2* and *Cxcl10* (Fig. 5E) were transiently upregulated at day 3 and returned toward baseline by day 7. Importantly, the magnitude of gene induction in the acute phase was comparable between virus groups. Together, these data demonstrate that skeletal muscle involvement during CVB3 infection is mild, transient, and independent of miR-1 detargeting.

### miR-1 detargeting abolishes CVB3-induced myocarditis

With systemic pathology preserved and no biologically relevant off-target effects, miR-1 detargeting establishes a heart-specific attenuation model. To quantify myocardial injury under these conditions, we first measured high-sensitivity troponin T (TnT) as a sensitive and specific serum marker of cardiomyocyte damage. Control-virus-infected mice exhibited a marked increase in TnT levels already at day 3 post infection, which was consistently elevated at day 7 at a magnitude comparable to day 3 (Fig. 6A). In striking contrast, TnT levels remained at baseline in CVB3-1-infected mice at both time points (Fig. 6A). Histopathological analysis corroborated these findings. At day 3 post infection, hearts from both groups showed only subtle alterations on H&E staining, characterized by occasional scattered immune cells, without formation of overt inflammatory foci (Fig. 6B). By day 7, however, control-virus-infected hearts displayed severe myocarditis with extensive focal and diffuse inflammatory infiltrates, widespread myocyte necrosis and pronounced tissue disruption. In sharp contrast, hearts from CVB3-1-infected mice showed intact myocardial architecture and a complete absence of inflammatory lesions or necrosis (Fig. 6B). To quantitatively define the inflammatory component of myocardial injury, we performed flow cytometric profiling of cardiac immune cell infiltrates (Supplementary Fig. S11–12 for gating strategy). At day 3 post infection, leukocyte numbers in the heart remained low and comparable between groups (Fig. 6C). By day 7, control-virus-infected hearts exhibited a massive accumulation of infiltrating myeloid immune cells (Fig. 6C, D), dominated by Ly6C^high^ monocytes, with a smaller contribution from dendritic cells (DC) and neutrophils (Fig. 6D). In contrast, no increase in cardiac leukocyte infiltration was observed in CVB3-1-infected mice, whose immune cell numbers remained at baseline levels (Fig. 6C, D). Lymphoid populations, including NK cells, T cells, and B cells, were present at low frequencies (Supplementary Fig. S4A–D).

Finally, we analyzed cardiac transcriptional responses. In control-virus-infected hearts, expression of *Ifn-β* and *Ifit3* was strongly induced at day 3 (Fig. 6E), reflecting local antiviral signaling. In CVB3-1-infected hearts, *Ifn-β* induction was already attenuated at this time point, whereas *Ifit3* expression remained comparable between groups. By day 7, both *Ifn-β* and *Ifit3* expression declined overall but remained significantly higher in control-virus-infected hearts compared with CVB3-1-infected hearts (Fig. 6E). Similarly, expression of pro-inflammatory cytokines (*Il-1β*, *Tnf-α*) (Fig. 6F) and chemokines (*Ccl2*, *Cxcl10*) (Fig. 6F) was comparable between groups at day 3. At day 7, however, control-virus-infected hearts maintained high expression of these inflammatory mediators, whereas levels were markedly reduced in CVB3-1-infected hearts (Fig. 6F,G), consistent with the absence of local viral replication and inflammation (Figs. 2A-B; 6B). In conclusion, miR-1-mediated detargeting enables functional uncoupling of systemic immune activation from cardiac pathology by suppressing cardiac viral replication while largely preserving systemic responses, thereby preventing overt myocarditis and allowing experimental dissection of myocardium-associated and systemic effects on cardiac dysfunction.

### CVB3 infection and myocardial injury jointly shape echocardiographic dysfunction

Leveraging this heart-specific attenuation model, we performed transthoracic echocardiography at baseline, during the acute phase (day 3), and during the subacute phase (day 7) following infection with CVB3-1 or the control virus CVB3-39 to quantify functional alterations and to assess how myocardial injury modifies echocardiographic readouts during CVB3 infection (Fig. 7A). We first analyzed CVB3-1-infected mice as a model of systemic infection without relevant myocardial viral replication or myocarditis. At day 3 post infection, cardiac output was significantly reduced compared with baseline (Table 1, Fig. 7B). This reduction was primarily driven by decreased left ventricular filling volumes (Table 1, Fig. 7C) and a corresponding reduction in stroke volume (Table 1, Fig. 7D), while ejection fraction remained preserved (Table 1, Fig. 7E). Consistent with reduced LV end-diastolic volumes, left ventricular end-diastolic diameter (LVIDd) was decreased (Table 1). Importantly, LV end-diastolic volume did not differ between CVB3-1- and CVB3-39-infected mice at day 3 (Table 3, Fig. 7C), suggesting that early reductions in ventricular filling are primarily driven by systemic effects of infection rather than myocardial involvement. Transmitral Doppler further supported impaired filling, with reduced mitral E-wave velocities during infection (Table 1, Fig. 7F).

**Table 3:** Between-group comparison of normalized echocardiographic parameters in CVB3-1– and CVB3-39–infected mice

% change from baseline	day 3		day 3: CVB3-1 vs. CVB3.39	day 7		day 7: CVB3-1 vs. CVB3.39	day 3 vs. day 7	
	CVB3-1	CVB3-39		CVB3-1	CVB3-39		CVB3-1	CVB3-39
LV Performance								
Heart rate [%]	104.8 ± 10.9	98.1 ± 6.9	ns	95.6 ± 10.1	103.4 ± 28.0	ns	*	ns
LVEDV [%]	74.4 ± 14.3	74.0 ± 14.0	ns	89.3 ± 17.0	69.1 ± 13.0	*	*	ns
LVESV [%]	68.4 ± 28.2	78.3 ± 27.2	ns	91.3 ± 23.1	64.8 ± 23.6	*	*	ns
Stroke volume [%]	82.3 ± 11.8	71.4 ± 14.3	ns	89.2 ± 21.4	76.2 ± 11.1	ns	ns	ns
Cardiac output [%]	86.0 ± 14.8	70.2 ± 15.1	*	84.7 ± 21.1	69.3 ± 12.7	*	ns	ns
EF [%]	113.2 ± 21.3	98.3 ± 21.8	ns	100.1 ± 15.2	113.4 ± 25.2	ns	ns	ns
M-Mode								
LVAWd [%]	111.8 ± 28.2	114.9 ± 27.1	ns	107.9 ± 26.6	130.8 ± 22.6	*	ns	ns
LVPWd [%]	109.8 ± 18.2	107.3 ± 17.8	ns	117.9 ± 26.0	129.1 ± 19.5	ns	ns	*
LVIDd [%]	87.6 ± 6.9	91.1 ± 7.1	ns	89.6 ± 5.3	83.1 ± 8.1	*	ns	*
LV Mass[%]	91.2 ± 20.8	99.0 ± 20.5	ns	97.4 ± 22.8	107.1 ± 16.9	ns	ns	ns
PW-Doppler								
MV E [%]	74.3 ± 14.0	66.5 ± 13.9	ns	77.0 ± 20.3	63.9 ± 7.1	ns	ns	ns
MV A [%]	81.1 ± 21.3	79.3 ± 12.1	ns	86.1 ± 33.2	61.7 ± 25.9	ns	ns	ns
MV E/A [%]	94.5 ± 17.1	83.4 ± 8.2	ns	97.4 ± 34.4	116.0 ± 41.3	ns	ns	*
IVRT [%]	100.9 ± 17.0	104.4 ± 9.9	ns	119.5 ± 28.2	124.8 ± 20.4	ns	ns	*
IVCT [%]	111.8 ± 32.2	106.0 ± 20.3	ns	136.4 ± 100.2	106.1 ± 35.5	ns	ns	ns
AET [%]	91.0 ± 12.6	99.4 ± 12.2	ns	92.2 ± 12.0	97.9 ± 8.6	ns	ns	ns
Tissue-Doppler								
MV E' [%]	86.3 ± 21.4	79.9 ± 30.3	ns	83.1 ± 25.6	73.0 ± 30.2	ns	ns	ns
MV A' [%]	76.9 ± 24.4	72.9 ± 26.8	ns	78.8 ± 29.1	57.0 ± 19.6	ns	ns	ns
MV E'/A' [%]	114.3 ± 52.1	121.0 ± 59.3	ns	132.1 ± 113.5	158.4 ± 124.6	ns	ns	ns
MV E/E' [%]	88.7 ± 16.1	93.3 ± 36.5	ns	104.1 ± 42.0	113.3 ± 63.8	ns	ns	ns
S' [%]	99.0 ± 16.3	90.3 ± 19.8	ns	92.0 ± 24.7	69.3 ± 25.0	ns	ns	ns
Strain analysis								
GLS [%]	91.3 ± 21.8	78.9 ± 13.5	ns	86.1 ± 14.7	80.5 ± 22.7	ns	ns	ns

C57BL/6J mice were infected intraperitoneally with 10^5^ PFU/mouse of CVB3-1 or CVB3-39. Transthoracic echocardiography was performed one day prior to infection (baseline) and at day 3 (acute phase) and day 7 (subacute phase) post infection immediately before sacrifice. Group sizes were as follows: baseline for d3 comparison: CVB3-1 (*N* = 12), CVB3-39 (*N* = 12); day 3: CVB3-1 (*N* = 12), CVB3-39 (*N* = 12); baseline for d7 comparison: CVB3-1 (*N* = 13), CVB3-39 (*N* = 11); day 7: CVB3-1 (*N* = 13), CVB3-39 (*N* = 11). Normalized data were used for statistical comparisons between virus strains at individual time points using unpaired two-tailed t-tests or Mann–Whitney U tests when normality was not met. Comparisons of the same virus strain across post-infection time points were performed on normalized data using unpaired two-tailed t-tests or Mann–Whitney U tests as appropriate. Statistical significance was defined as *p* ≤ 0.05 and is indicated by a single asterisk (*)

Despite comparable filling volumes, cardiac output was already significantly lower in CVB3-39-infected mice at day 3 in comparison to CVB3-1 infected mice (Table 3, Fig. 7B), indicating an additional functional deficit associated with early myocardial injury. This group difference between CVB3-1 and CVB3-39-infected mice persisted at day 7 (Table 3, Fig. 7B). In both virus groups, cardiac output remained reduced at a level similar to that observed during the acute phase (Tables 1, 2, 3), with CVB3-39 showing a consistently greater reduction than CVB3-1 at both time points (Table 3, Fig. 7B). Between day 3 and day 7, modest differences in diastolic filling emerged between groups: while filling volumes remained reduced compared with baseline in both groups (Tables 1, 2), LV end-diastolic volumes were higher in CVB3-1-infected mice than in CVB3-39-infected mice at day 7 (Table 3, Fig. 7C). However, stroke volume and cardiac output remained suppressed in both groups (Table 3, Fig. 7D,B), and ejection fraction stayed preserved and comparable between groups throughout (Table 3, Fig. 7E).

Tissue Doppler analysis of early diastolic myocardial velocities (E′) in CVB3-1-infected mice showed only modest reductions with substantial inter-individual variability at days 3 and 7 (Table 1, Fig. 7G**,**H). In contrast, CVB3-39 infection was associated with a more pronounced decline in E′, already evident at day 3 and persisting at day 7 (Table 2, Fig. 7G,H). The E/E′ ratio remained unchanged following infection and did not differ between virus strains (Tables 1, 2, 3, Fig. 7I). Similarly, tissue Doppler S′ showed no significant differences between virus groups, although a reduction relative to baseline was observed in CVB3-39-infected mice at day 7, again with marked inter-individual variability (Tables 2, Fig. 7J). Thus, while transmitral inflow and tissue Doppler indices reflect infection-associated alterations in filling and myocardial motion, their variability and limited discriminatory power restrict robust myocarditis-specific interpretation. Analysis of deformation parameters revealed that global longitudinal strain (GLS) was largely preserved during infection with CVB3-1 at day 3 (Table 1, Fig. 7K**,**L), whereas CVB3-39-infected mice exhibited a reduction at this time point (Table 2, Fig. 7K,L). By day 7, GLS was reduced in both groups (Tables 1, 2),, Fig. 7K,L), and no significant differences between virus strains were detectable (Table 3), suggesting that GLS changes are not myocarditis-specific in the subacute phase of this model.

Overall, CVB3-1 infection was associated with an early reduction in cardiac output accompanied by decreased LV end-diastolic volume and stroke volume, consistent with reduced ventricular filling, while ejection fraction remained unchanged. In CVB3-39-infected mice, cardiac output was further reduced despite comparable LVEDV, suggesting an additional functional impairment associated with myocardial injury. Although global longitudinal strain was transiently lower in CVB3-39 during the acute phase, deformation parameters and tissue Doppler indices did not provide consistent discrimination between virus strains in the subacute phase.

## Discussion

Preclinically, echocardiography is widely used to assess cardiac function in murine models of coxsackievirus B3 myocarditis [2, 33, 51, 52], one of the most commonly employed experimental systems to study virus–host interactions in the heart [27]. In this context, functional alterations are frequently interpreted as direct surrogates of myocardial injury and inflammation. The present study addresses a key methodological limitation of this approach by demonstrating that echocardiographic dysfunction develops in the setting of systemic infection. Using a microRNA-guided viral detargeting strategy to selectively suppress cardiac viral replication while largely preserving systemic infection, we provide direct experimental evidence that reductions in cardiac output and associated echocardiographic abnormalities occur in the absence of cardiomyocyte injury and histological myocarditis. At the same time, myocardial involvement contributes an additional functional component that is detectable early in infection. These findings establish that echocardiographic readouts in this model reflect a composite phenotype arising from both systemic and myocardial processes.

Selective cardiac attenuation was achieved using microRNA-guided viral detargeting [6], a strategy that enables tissue-specific regulation of viral replication. In our model, miR-1-mediated detargeting effectively suppressed cardiac viral replication while largely preserving systemic infection kinetics and disease manifestations, with only minor differences observed at selected time points. Minor reductions in viral burden in non-cardiac tissues during the subacute phase likely reflect altered viral kinetics [17] and were not associated with relevant changes in tissue pathology or systemic inflammatory responses. Importantly, pancreatic and hepatic involvement, systemic cytokine responses, and overall disease severity were broadly comparable between groups, suggesting that the absence of myocarditis in CVB3-1 infection is unlikely to be explained by global attenuation of viral replication. CVB3-1 infection maintained physiological troponin T levels at days 3 and 7 despite low-level cardiac viral presence, consistent with residual replication remaining below the threshold for relevant myocytolysis. In contrast, unrestricted cardiac replication in CVB3-39 infection resulted in early troponin T elevation preceding leukocyte infiltration, followed by sustained myocardial injury with overt inflammatory myocarditis. Consistent with the established concept that endogenous microRNAs can be exploited to regulate viral replication or transgene expression in a tissue-selective manner [38, 39], this experimental configuration enabled direct separation of systemic infection from myocardial viral replication and thereby provides a framework to dissect cardiac-specific consequences of viral infection within the context of an otherwise preserved systemic response.

A central finding of this study is that echocardiographic dysfunction during CVB3 infection reflects two mechanistically distinct components: an early impairment associated with systemic infection and an additional myocardial injury-related component. Reduced cardiac output was established early, occurred in the absence of detectable myocardial injury, and was accompanied by decreased ventricular filling volumes while ejection fraction remained preserved during infection with both CVB3-1 and CVB3-39. This pattern is consistent with alterations in ventricular loading conditions during infection, with systemic inflammatory responses contributing to these changes. Proinflammatory cytokines such as TNF-α and IL-1β are established mediators of myocardial inflammation in CVB3 myocarditis [10, 29], and are associated with progression to autoimmune myocarditis [41]. Both cytokines were robustly induced during the acute phase of infection in both experimental groups and have been associated with hemodynamic alterations in experimental settings [34]. Elevated circulating cytokine levels have been linked to changes in vascular tone and circulatory regulation, which may influence cardiac preload and thereby modulate cardiac output. In this context, cytokine-induced nitric oxide signaling represents a plausible mechanism, as TNF-α and IL-1β can stimulate inducible nitric oxide synthase and promote vasodilation [5, 26]. However, direct hemodynamic measurements were not performed in the present study, and the contribution of altered vascular resistance or preload cannot be quantified. Notably, these considerations align with the broader concept that cardiac performance is closely linked to systemic vascular regulation, whereby changes in vascular tone and resistance can substantially influence cardiac loading conditions [31].

Beyond these systemic effects, myocardial injury appears to contribute an additional functional component. At day 3, cardiac output was further reduced in control-virus-infected mice despite comparable end-diastolic ventricular volume, suggesting a contribution of early myocardial involvement. This impairment was partially reflected in altered global longitudinal strain (GLS), although substantial variability limited its discriminatory value in the subacute phase. Mechanistically, these early functional alterations are most plausibly attributable to direct viral cytopathic injury. Active cardiomyocyte infection promotes sarcolemmal disruption and myofibrillar damage [32], reflected by early troponin release [7]. While viral replication can also trigger innate immune signaling [3], myocardial cytokine expression did not differ substantially between groups at this stage, supporting a predominant contribution of direct viral injury. By day 7, inflammatory signaling diverged more clearly between groups; however, functional impairment did not scale with the extent of histological myocarditis. Despite marked inflammation and sustained troponin elevation, cardiac output was not further reduced, indicating a dissociation between myocardial injury and functional readouts at this stage. Sustained inflammatory signaling may contribute to myocardial dysfunction through effects on contractility and cellular signaling pathways [34], although these mechanisms likely represent one component of a multifactorial process. In this regard, extra-cardiac organs such as spleen, which serves as a major reservoir of innate immune cells during systemic inflammatory states [16], might contribute to the circulating cytokine milieu observed. Complementary CVB3 infection studies suggest that myocardial inflammation alone may not be sufficient to cause marked global dysfunction [38], and prior reports on ejection fraction in CVB3 myocarditis have been inconsistent [2, 21, 25, 33, 42]. In this context, our data underscore that preserved ejection fraction can coexist with marked functional impairment, particularly when altered loading conditions and intrinsic myocardial injury overlap. The broader relevance of our findings is supported by evidence from other viral infections. In SARS-CoV-2 and hepatitis C virus infection, indirect immune-mediated mechanisms, including cytokine storm and TNF-α–driven nitric oxide dysregulation, have been implicated in cardiac dysfunction even in the absence of direct myocardial infection [45]. In contrast, an influenzavirus study using miRNA-guided cardiac detargeting demonstrated that reduced cardiac viral replication attenuates myocardial fibrosis and electrical dysfunction despite preserved systemic lung inflammation [23]. Notably, cardiac function in this study was assessed by electrocardiography rather than echocardiography, leaving the impact of systemic infection on hemodynamic function unresolved.

Taken together, our findings demonstrate that in the murine CVB3 myocarditis model, echocardiographic alterations reflect a composite phenotype arising from both systemic infection and myocardial injury. Reduced cardiac output develops early in the course of infection and persists despite marked differences in myocardial inflammation, indicating that functional impairment does not directly scale with the extent of myocarditis. These results do not challenge the presence of myocardial dysfunction in viral myocarditis but emphasize that functional readouts cannot be interpreted in isolation from systemic disease processes. Accordingly, accurate assessment of cardiac phenotypes in preclinical infectious disease models requires integration of functional data with histological, immunological, and virological analyses, considering cross-organ and temporal dynamics.

## Supplementary Information

Below is the link to the electronic supplementary material.

Supplementary file1 (DOCX 7269 KB)

## Data Availability

All data reported in this article are available from the corresponding author upon reasonable request.

## Abbreviations

AET: Aortic ejection time
ALT: Alanine aminotransferase
AST: Aspartate aminotransferase
CCL2: C–C motif chemokine ligand 2
CMR: Cardiovascular magnetic resonance
CPE: Cytopathic effect
CVB3: Coxsackievirus B3
CXCL10: C-X-C motif chemokine ligand 10
DCM: Dilated cardiomyopathy
DCs: Dendritic cells
ECG: Electrocardiogram
ECLIA: Electrochemiluminescence immunoassay
eCM: Embryonal cardiomyocytes
EF: Ejection fraction
EMB: Endomyocardial biopsy
ESC: European Society of Cardiology
FEM: Forschungseinrichtungen für Experimentelle Medizin
GLS: Global longitudinal strain
HPRT: Hypoxanthine–guanine phosphoribosyltransferase
hsTNT: High sensitivity Troponin T
IFIT3: Interferon-Induced Protein with Tetratricopeptide Repeats 3
IFN-α: Interferon alpha
IFN-β: Interferon beta
IL-1β: Interleukin 1 beta
IL-6: Interleukin 6
ISG: Interferon stimulated genes
IVCT: Isovolumetric contraction time
IVRT: Isovolumetric relaxation time
iWAT: Inguinal white adipose tissue
KC: Kupffer cells
LAGeSo: Landesamt für Gesundheit und Soziales Berlin
LDH: Lactate dehydrogenase
LV Mass: Left ventricular mass
LVAWd: Left ventricular anterior wall in diastole
LVEDV: Left ventricular end-diastolic volume
LVEF: Left ventricular ejection fraction
LVESV: Left ventricular end-systolic volume
LVIDd: Left ventricular internal diameter in diastole
LVPWd: Left ventricular posterior wall in diastole
m. gastrocnemius: Musculus gastrocnemius
m. quadriceps fem.: Musculus quadriceps femoris
MHC: Mayor histocompatibility complex
miR: MicroRNA
miR-TS: MicroRNA target site
MIS: Muscle inflammation score
MoMF: Monocyte-derived macrophages
MV: Mitral valve
NK cells: Natural killer cells
T cells: Thymus cells
TNF-α: Tumor necrosis factor alpha
VP: Viral protein
