# Supplementary material for: Dissecting myocardial and systemic drivers of cardiac dysfunction in the murine CVB3 myocarditis model using microRNA-guided viral detargeting
Source: Basic Res Cardiol. 2026 May 29;121(4):775–99. doi: 10.1007/s00395-026-01187-4 (PMC13372866; doi:10.1007/s00395-026-01187-4)
Supplement: Supplementary file 1 — Supplementary file1 (DOCX 7269 KB) [file 395_2026_1187_MOESM1_ESM.docx]

**SUPPLEMENTARY FILE**

**Dissecting systemic and myocardial contributions to echocardiographic dysfunction in experimental viral myocarditis using cardiac microRNA-guided viral attenuation**

Sarah Ochs^1^, Sandra Pinkert^1,2^, Sophia Borowski^1,2^, Lisa GM Huis in ‘t Veld^1^, Nicolas Kelm^1,10^, Anja Geisler^4^, Henry Fechner^4^, Ziya Kaya^5,6^, Anne Hausen^7^, Karin Klingel^3^, Matthias M. Gaida^7,8,9^, Antje Beling^1,2*^

1 – Charité – Universitätsmedizin Berlin, corporate member of Freie Universität Berlin and Humboldt-Universität zu Berlin, Institute of Biochemistry, 10117 Berlin, Germany

2 – Deutsches Zentrum für Herz-Kreislauf-Forschung, partner site Berlin, 10117 Berlin, Germany

3 – Cardiopathology, Institute for Pathology and Neuropathology, University Hospital Tübingen, 72076 Tübingen, Germany

4 – Department of Applied Biochemistry, Institute of Biotechnology, Technische Universität Berlin, 15533 Berlin, Germany

5 – Kardiologie, Angiologie und Pneumologie, Medizinische Klinik für Innere Medizin III, Universitätsklinikum Heidelberg, 69120 Heidelberg, Germany.

6 – Deutsches Zentrum für Herz-Kreislauf-Forschung (DZHK), Partner Site Heidelberg, 69120 Heidelberg, Germany.

7 – Institute of Pathology, University Medical Center Mainz, Johannes-Gutenberg-Universität Mainz, 55131 Mainz, Germany

8– TRON, Translational Oncology at the University Medical Center of the Johannes Gutenberg University Mainz, Mainz, Germany

9 – Research Center for Immunotherapy, University Medical Center Mainz, Johannes-Gutenberg-Universität Mainz, 55131 Mainz, Germany

10 – Department of Medicine I, Department of Gastroenterology and Hepatology, Faculty of Medicine and University Hospital Carl Gustav Carus, TUD Dresden University of Technology, 01069 Dresden, Germany.

^*^**Corresponding author**

Prof. Antje Beling

Phone: 0049 30 450 528 187

Email: antje.beling@charite.de

Charité – Universitätsmedizin Berlin

Institute of Biochemistry

Charitéplatz 1

10117 Berlin

Germany

ORCID ID: 0000-0002-1826-5248


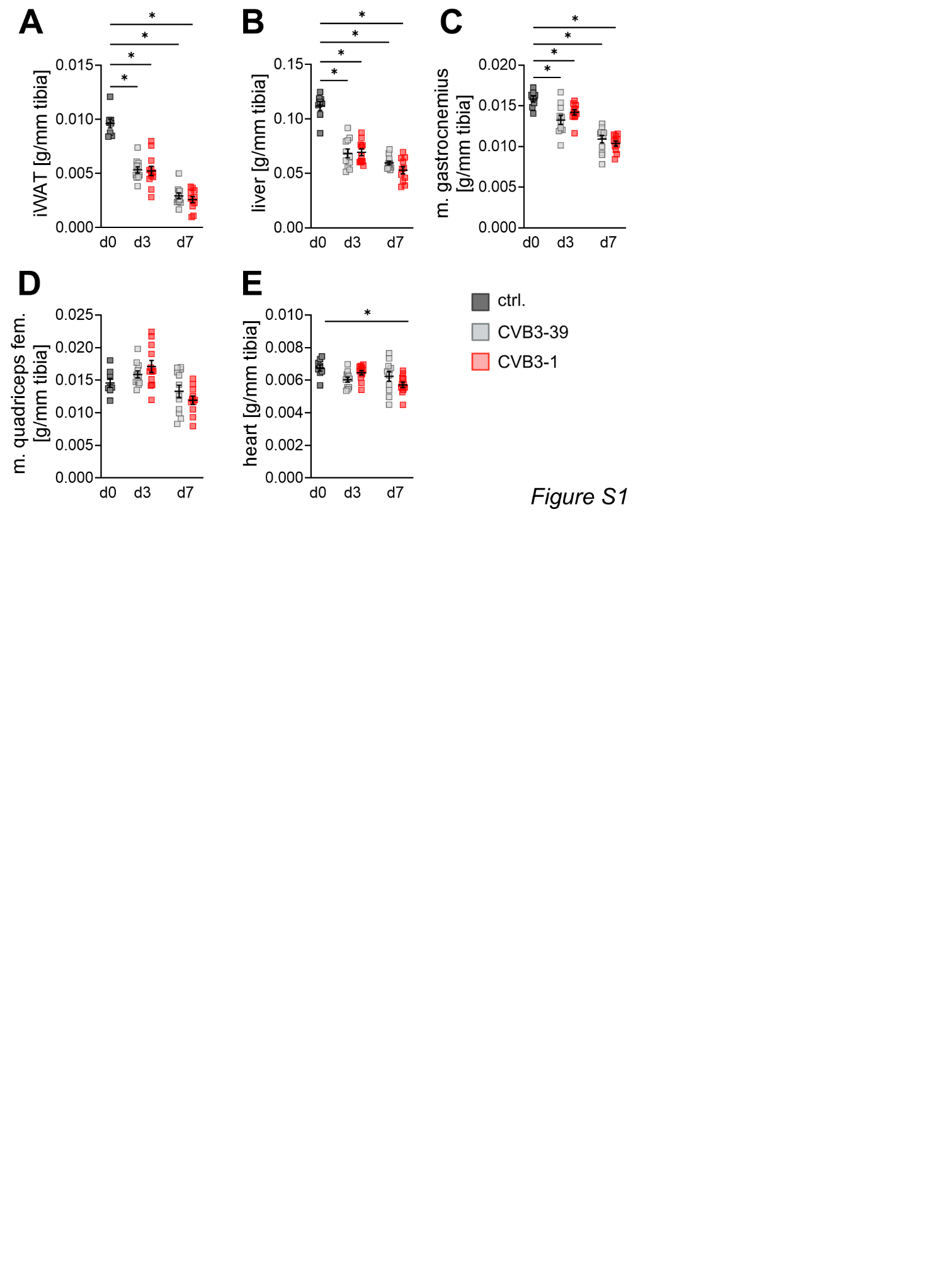


**Fig.S1 Organ weight reduction is indistinguishable in both virus infections.** C57BL/6J were intraperitoneally infected with 10^5^ PFU of CVB3-1 or CVB3-39 and analyzed at day 3 (acute phase) and day 7 (subacute phase). Age-matched uninfected mice served as baseline controls (day 0). Group sizes: d0 (N = 8), d3 CVB3-39 (N = 12), d3 CVB3-1 (N = 12), d7 CVB3-39 (N = 12), d7 CVB3-1 (N = 13). (**A-E**) Organs were weighed and the organ weight was normalized to the tibia length for (**A**) inguinal white adipose tissue (iWAT), (**B**) liver, (**C**) musculus gastrocnemius (m. gastrocnemius), (**D**) musculus quadriceps femoris (m. quadriceps fem.) and (**E**) heart. Data are mean ± SEM. Outliers were detected using the ROUT (Q=1%) method (A-E). Two-way ANOVA with Sidak's post hoc test was used for datasets with two independent factors (time and virus strain) (A-E). One-way ANOVA with Dunnett's post hoc test compared post-infection time points to baseline (A-E)


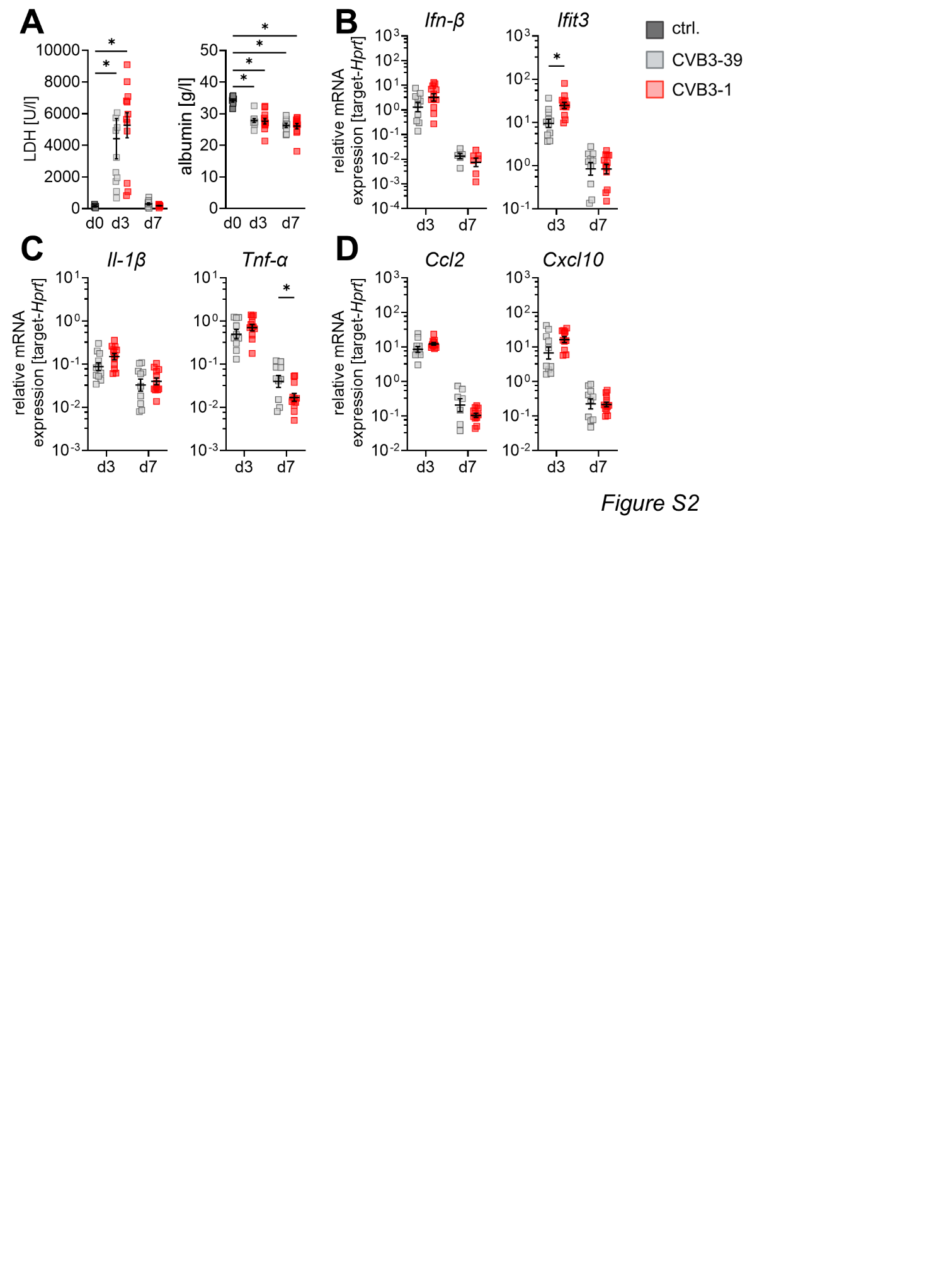


**Fig.S2 Liver pathology shows similar severity of infection in both viral strains.** C57BL/6J were intraperitoneally infected with 10^5^ PFU of CVB3-1 or CVB3-39 and analyzed at day 3 (acute phase) and day 7 (subacute phase). Age-matched uninfected mice served as baseline controls (day 0). Group sizes: d0 (N = 8), d3 CVB3-39 (N = 12), d3 CVB3-1 (N = 12), d7 CVB3-39 (N = 12), d7 CVB3-1 (N = 13). (**A**) Serum was analyzed for lactate dehydrogenase (LDH) and albumin levels by a veterinary lab. (**B-D**) mRNA expression of interferon/interferon-stimulated genes (IFN/ISG) (**B**), cytokines (**C**), and chemokines (**D**) in liver tissue measured by RT-qPCR. Data are mean ± SEM. Outliers were detected using the ROUT (Q=1%) method (A-D). Two-way ANOVA with Sidak's post hoc test was used for datasets with two independent factors (time and virus strain) (A-D). One-way ANOVA with Dunnett's post hoc test compared post-infection time points to baseline (A)


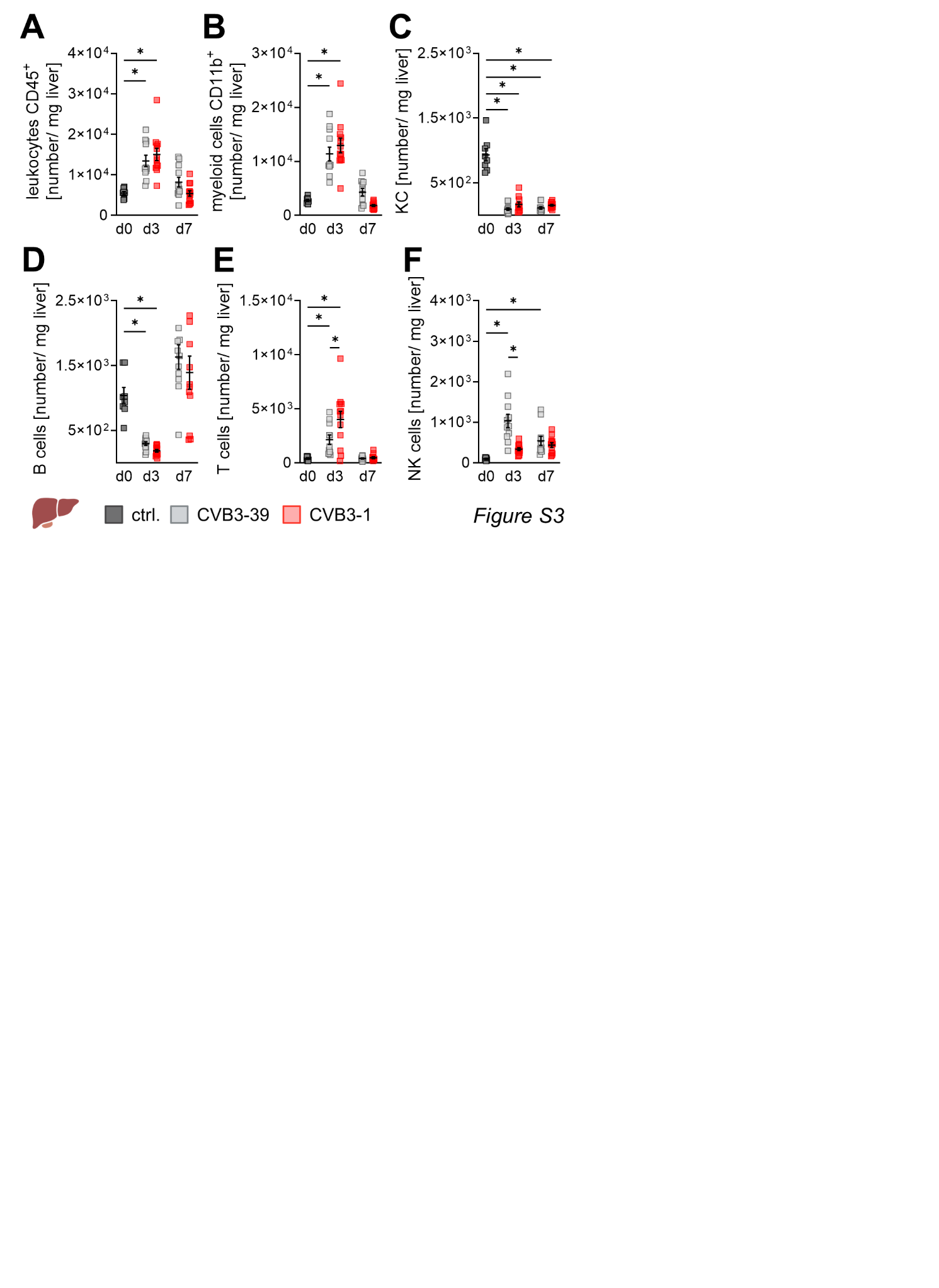


**Fig.S3 Transient myeloid infiltration dominates the acute immune response while lymphoid cells play a minor role.** C57BL/6J were intraperitoneally infected with 10^5^ PFU of CVB3-1 or CVB3-39 and analyzed at day 3 (acute phase) and day 7 (subacute phase). Age-matched uninfected mice served as baseline controls (day 0). Group sizes: d0 (N = 8), d3 CVB3-39 (N = 12), d3 CVB3-1 (N = 12), d7 CVB3-39 (N = 12), d7 CVB3-1 (N = 13). (**A-F**) Immune cell populations were analyzed by flow cytometry and cell numbers per milligram of liver tissue are shown for the following populations: (**A**) CD45^+^ leukocytes, (**B**) CD45^+^lineage^-^CD11b^+^ myeloid cells, (**C**) CD45^+^lineage^-^CD11b^intermediate^Ly6G^-^F4/80^high^ Kupffer cells (KC) with lineage markers CD3^-^B220^-^Ter119^-^CD49b^-^. Additionally, following lymphoid populations are shown: (**D**) CD45^+^CD19^+^B220^+^ B-cells, (**E**) CD45^+^CD19^-^CD3^+^ T cells, (**F**) CD45^+^CD19^-^CD3^-^NK1.1^+^ NK cells. Data are mean ± SEM. Outliers were detected using the ROUT (Q=1%) method (A-F). Two-way ANOVA with Sidak's post hoc test was used for datasets with two independent factors (time and virus strain) (A-F). One-way ANOVA with Dunnett's post hoc test compared post-infection time points to baseline (A-F)


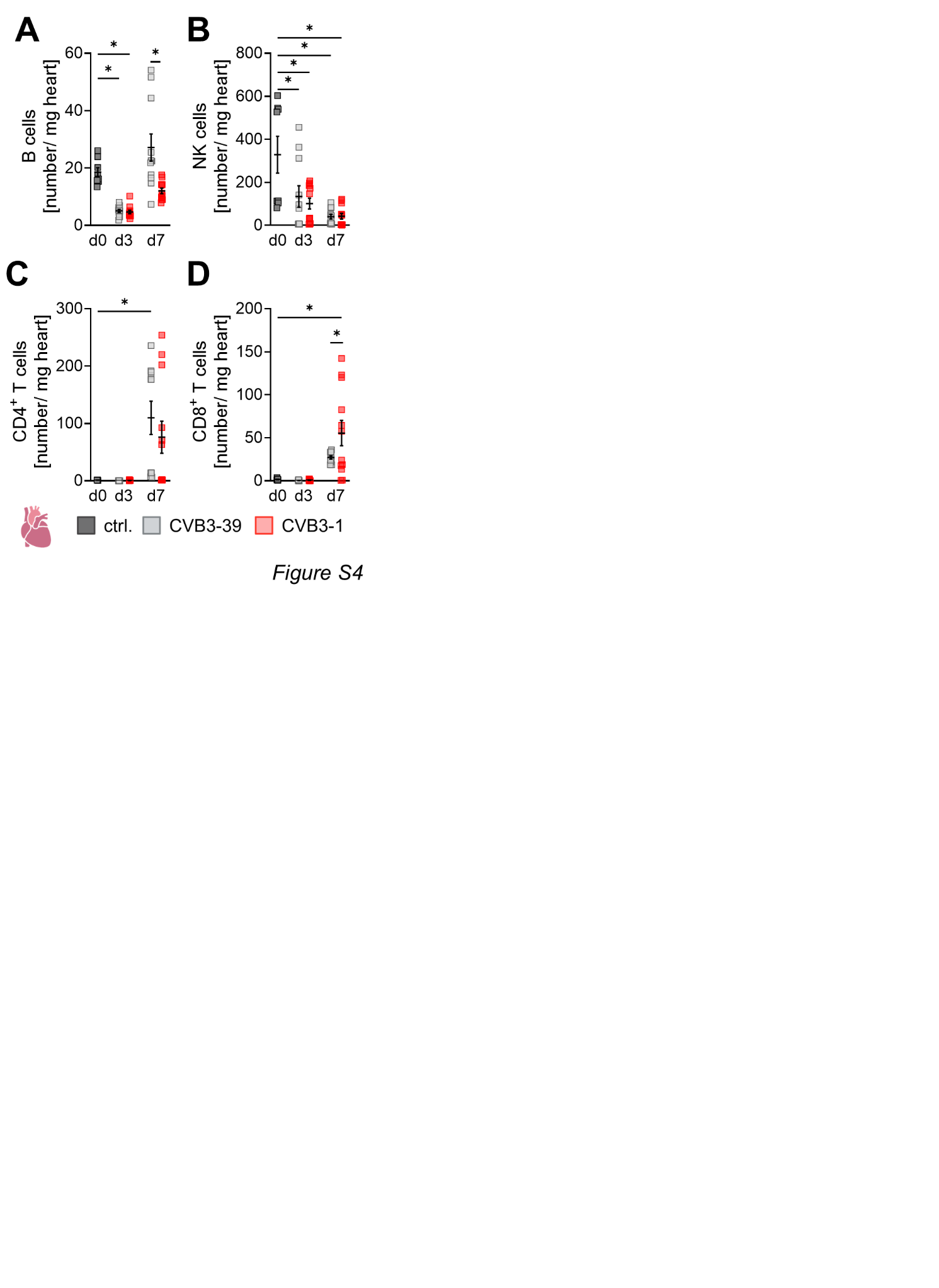


**Fig.S4 Minor lymphoid cell infiltration in heart after virus infection.** C57BL/6J were intraperitoneally infected with 10^5^ PFU of CVB3-1 or CVB3-39 and analyzed at day 3 (acute phase) and day 7 (subacute phase). Age-matched uninfected mice served as baseline controls (day 0). Group sizes: d0 (N = 8), d3 CVB3-39 (N = 12), d3 CVB3-1 (N = 12), d7 CVB3-39 (N = 12), d7 CVB3-1 (N = 13). (**A-D**) Immune cell populations were analyzed by flow cytometry and cell numbers per milligram of heart tissue are shown for the following populations: (**A**) CD45^+^CD11b^-^NK1.1^-^B220^+^CD3^-^ B-cells, (**B**) CD45^+^CD11b^-^B220^+^CD3^-^NK1.1^+^ NK cells, (**C**) CD45^+^CD11b^-^B220^-^CD3^+^CD4^+^CD8^-^ T helper cells, (**D**) CD45^+^CD11b^-^B220^-^CD3^+^CD4^-^CD8^+^ cytotoxic T lymphocytes. Data are mean ± SEM. Outliers were detected using the ROUT (Q=1%) method (A-D). Two-way ANOVA with Sidak's post hoc test was used for datasets with two independent factors (time and virus strain) (A-D). One-way ANOVA with Dunnett's post hoc test compared post-infection time points to baseline (A-D)


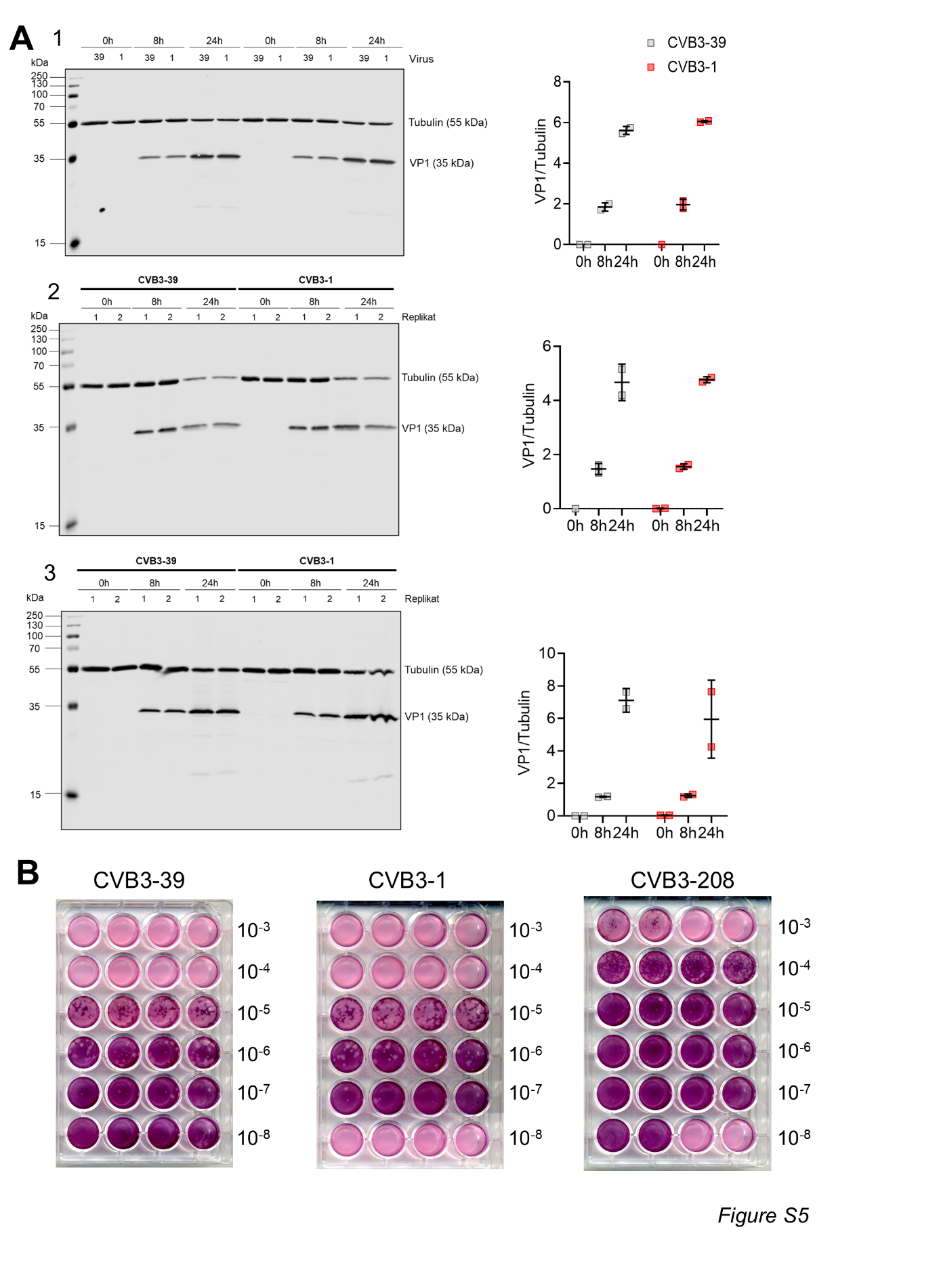


**Fig.S5 Uncropped western blot and plaque assay images.** HeLa cells were infected with MOI 1 of CVB3-1 and CVB3-39 and harvested after 0, 8 and 24h. (**A**) VP1 protein expression was analyzed by Western blot using tubulin as a loading control. Uncropped pictures of all three repetitions with the corresponding densitometric quantification are shown for **Fig.1F**. (**B**) Uncropped pictures of the representative plaque assay are shown for **Fig.1C**


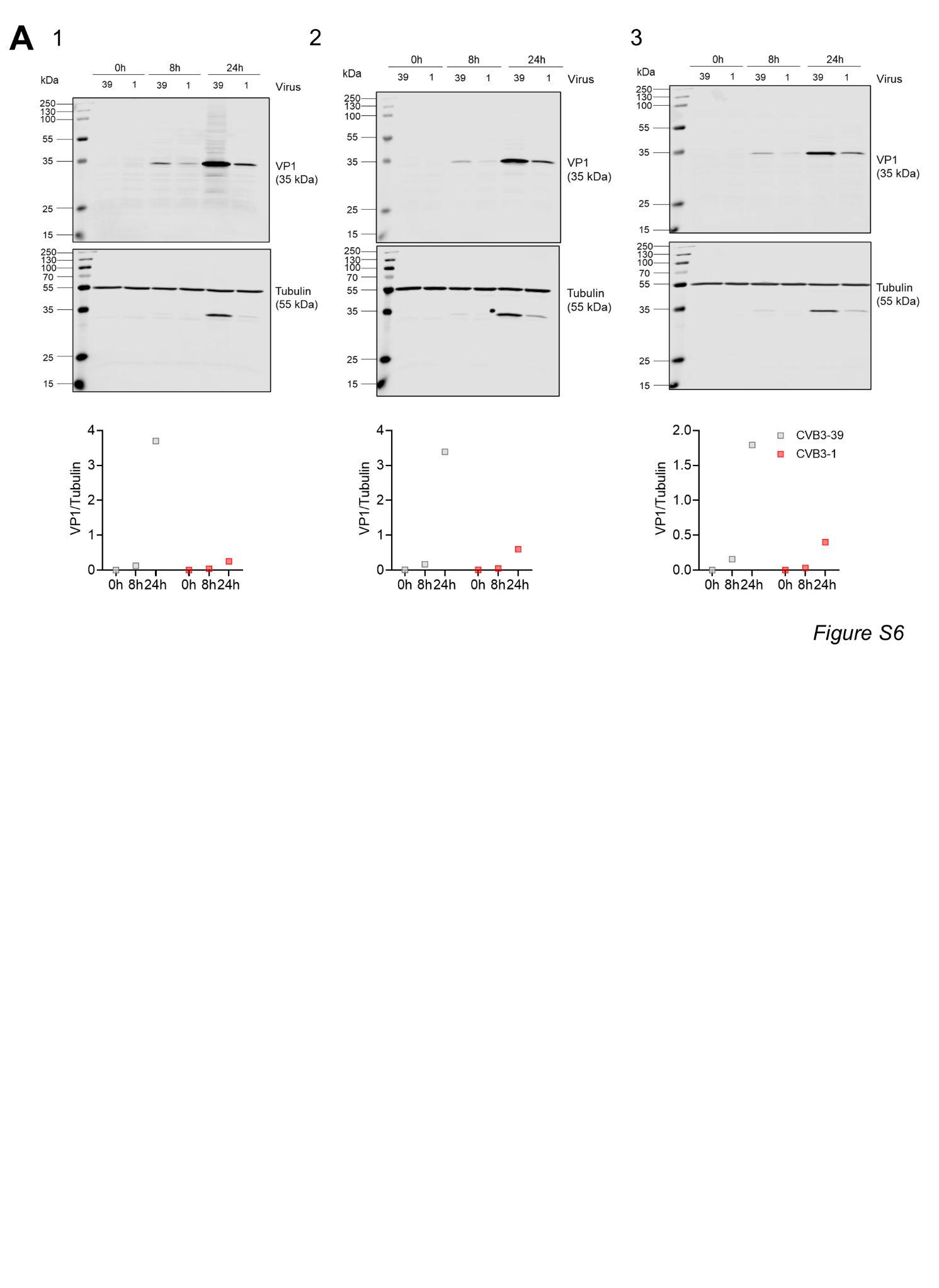


**Fig.S6 Uncropped western blot images.** Embryonal cardiomyocytes (eCM) were infected with MOI 5 of CVB3-1 or CVB3-39 and harvested after 0, 8 and 24h. (**A**) VP1 protein expression was analyzed by Western blot using tubulin as a loading control. Uncropped pictures of all three repetitions with the corresponding densitometric quantification are shown for **Fig.1J**


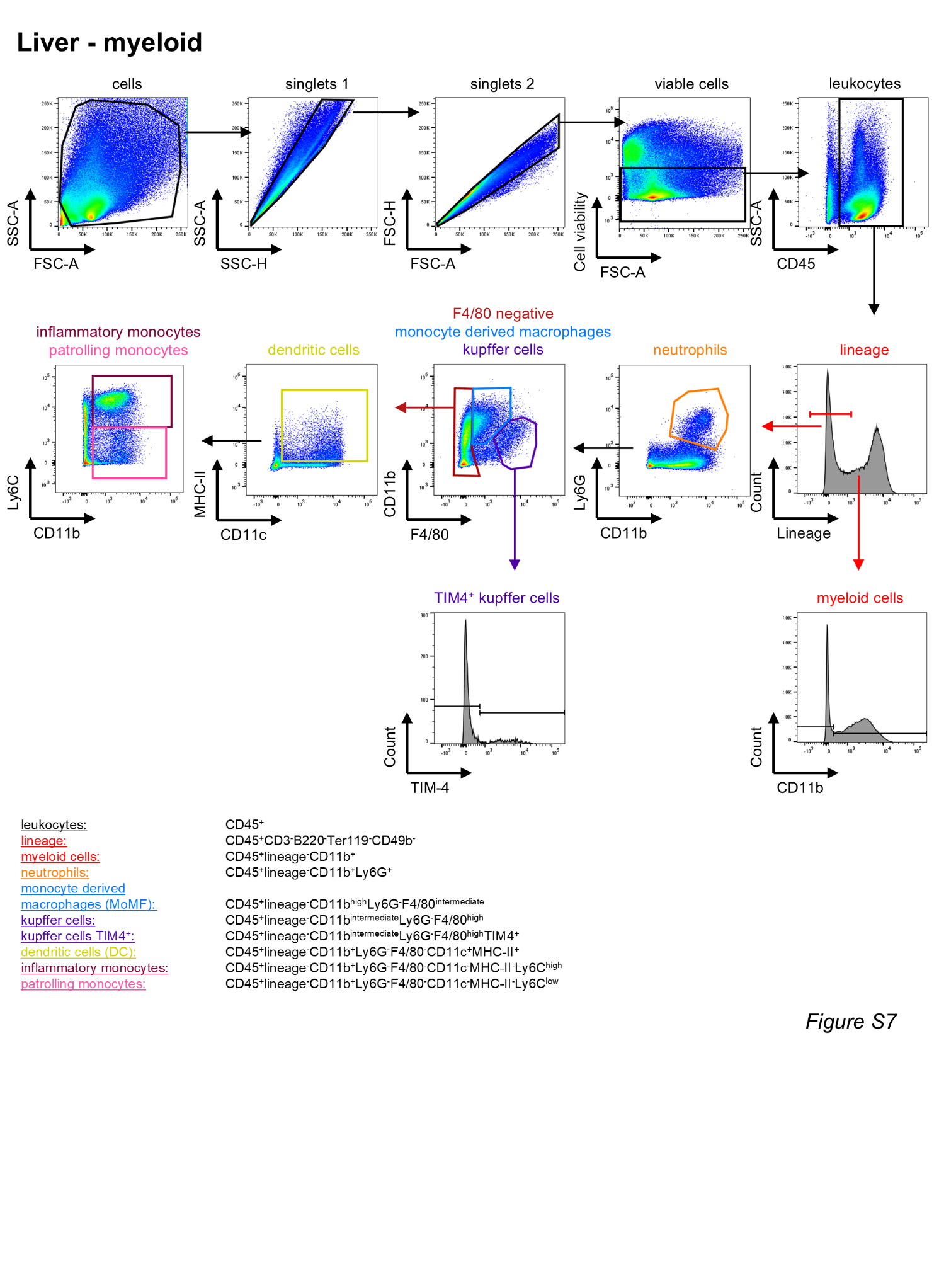


**Fig.S7 Representative gating strategy for quantifying myeloid immune cells in the liver during infection by flow cytometry.** To characterize myeloid immune cells from liver tissue, cells were stained with a panel of fluorochrome-conjugated antibodies targeting lymphoid and myeloid surface markers and analyzed by flow cytometry. Debris and cell doublets were first excluded using FSC-A/SSC-A, SSC-A/SSC-H, and FSC-A/FSC-H gating. Dead cells were then removed based on staining with viability dye. Then, leukocytes were selected and further refined by removing lymphoid and non-myeloid cells based on lineage marker exclusion. Subsequently, specific myeloid cell subsets were identified according to specific marker combinations. The gating parameters for each immune cell population are described in detail below the representative gating plots


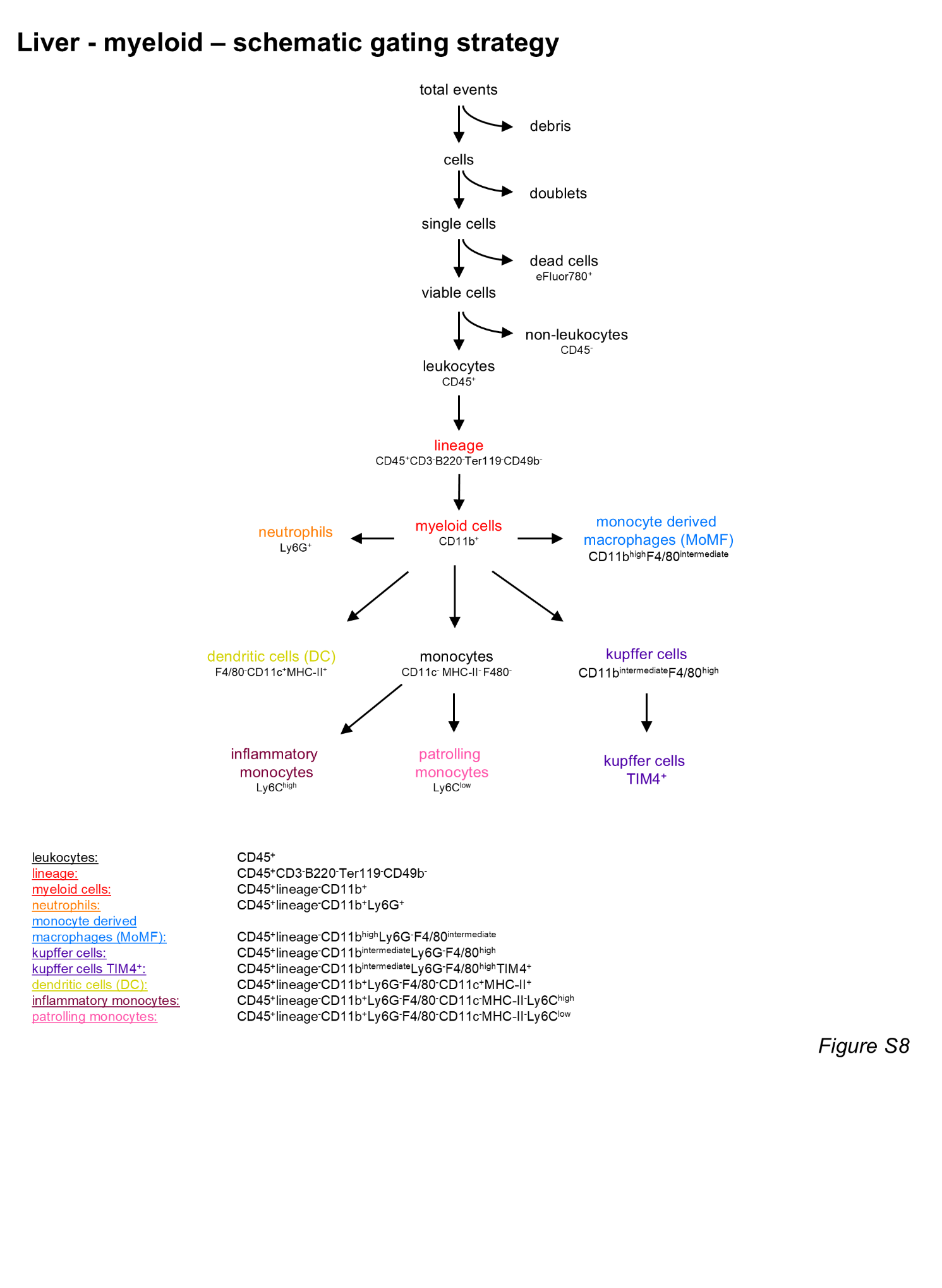


**Fig.S8 Schematic overview of the flow cytometry gating strategy for myeloid immune cells in the liver**. This scheme illustrates the gating strategy applied in the flow cytometry analysis summarizing the representative gating plot presented in Figure S10. The gating parameters for each immune cell population are indicated below the schematic overview


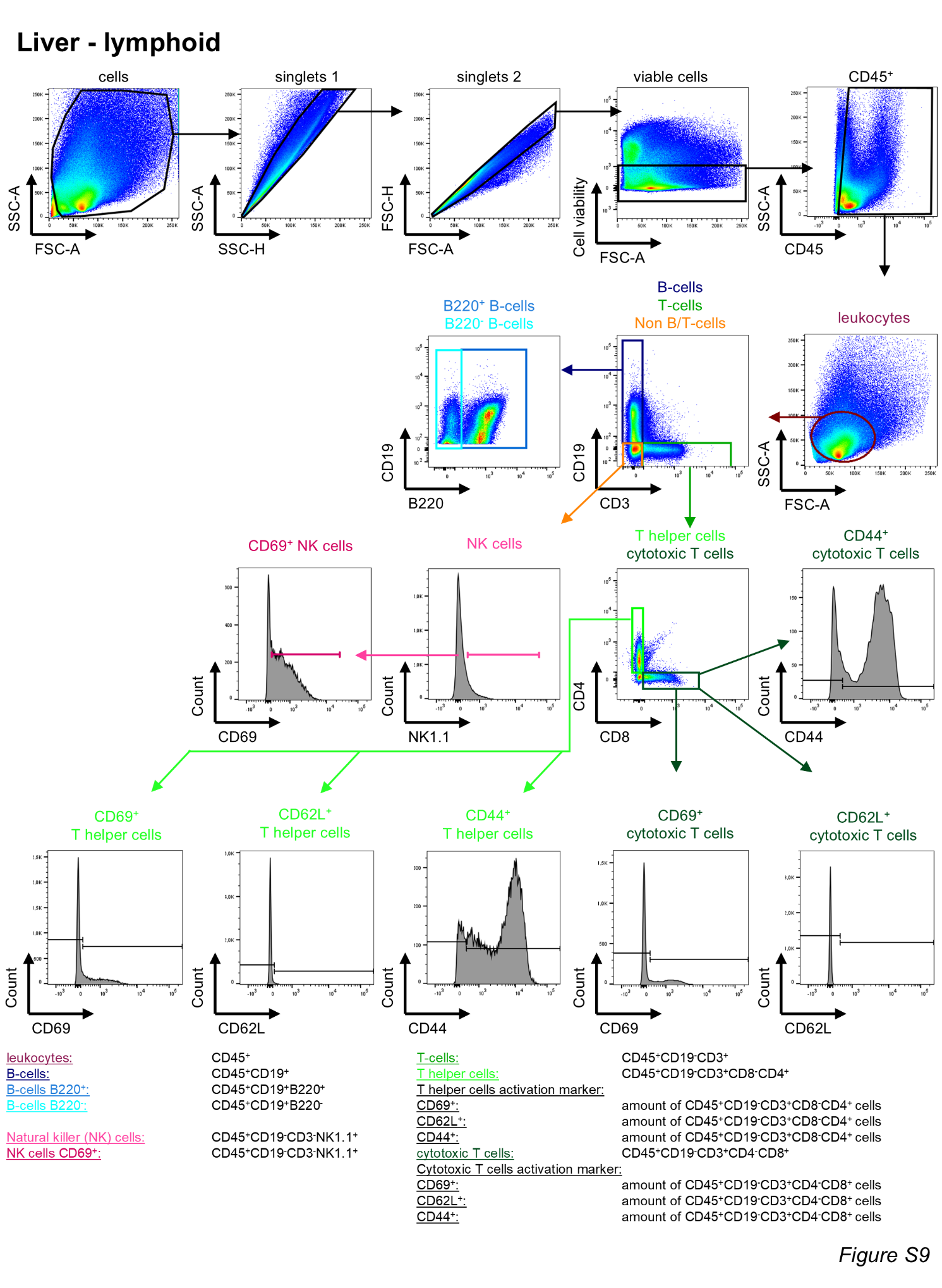


**Fig.S9 Representative gating strategy for quantifying lymphoid immune cells in the liver during infection by flow cytometry.** To characterize lymphoid immune cells from liver tissue, cells were stained with a panel of fluorochrome-conjugated antibodies targeting lymphoid surface markers and analyzed by flow cytometry. Debris and cell doublets were first excluded using FSC-A/SSC-A, SSC-A/SSC-H, and FSC-A/FSC-H gating. Dead cells were then removed based on staining with viability dye. Subsequently, leukocytes were selected, and distinct lymphoid subsets were identified according to specific marker combinations. The gating parameters for each immune cell population are described in detail below the representative gating plots


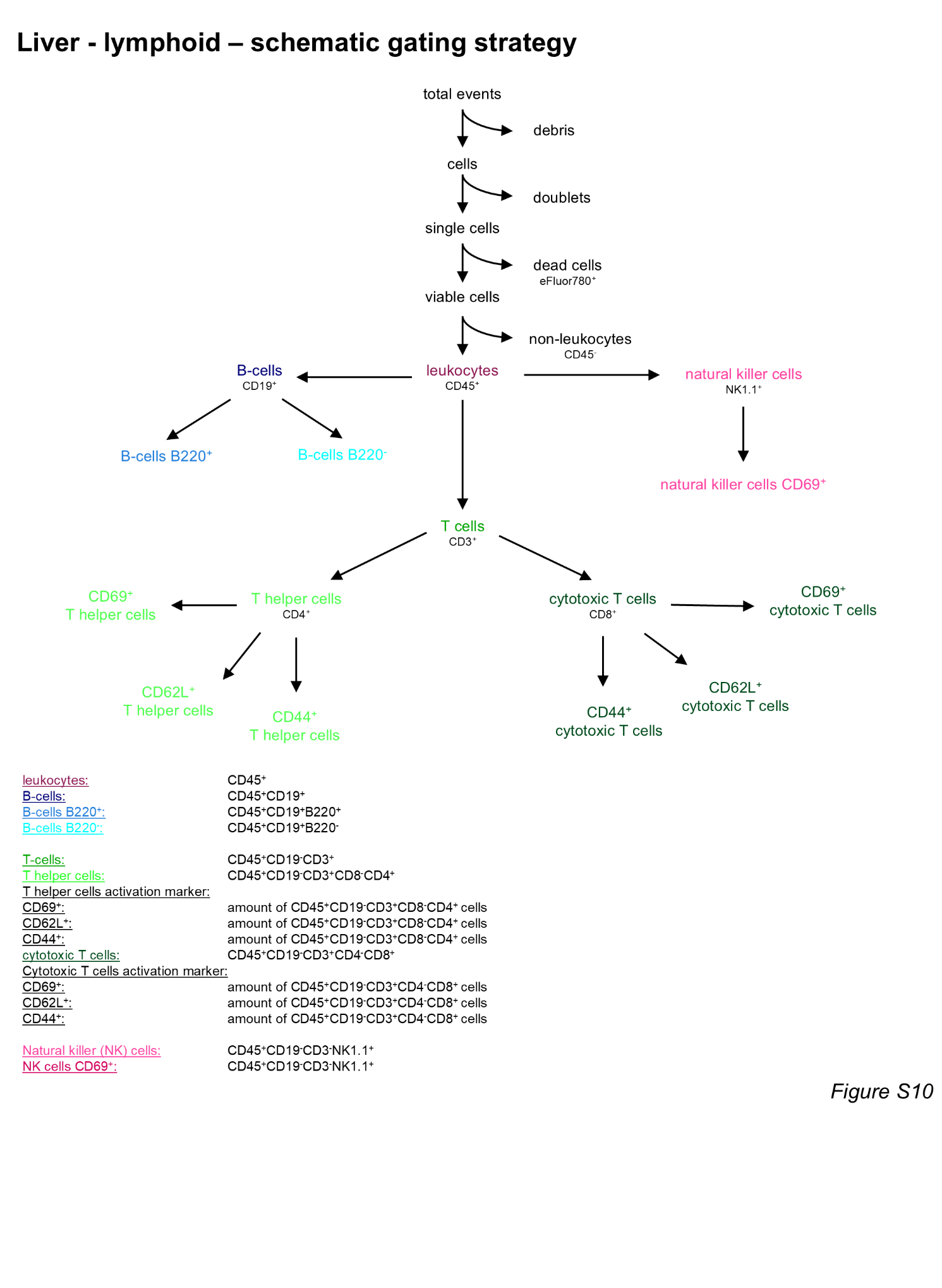


**Fig.S10 Schematic overview of the flow cytometry gating strategy for lymphoid immune cells in the liver**. This scheme illustrates the gating strategy applied in the flow cytometry analysis summarizing the representative gating plot presented in Figure S8. The gating parameters for each immune cell population are indicated below the schematic overview


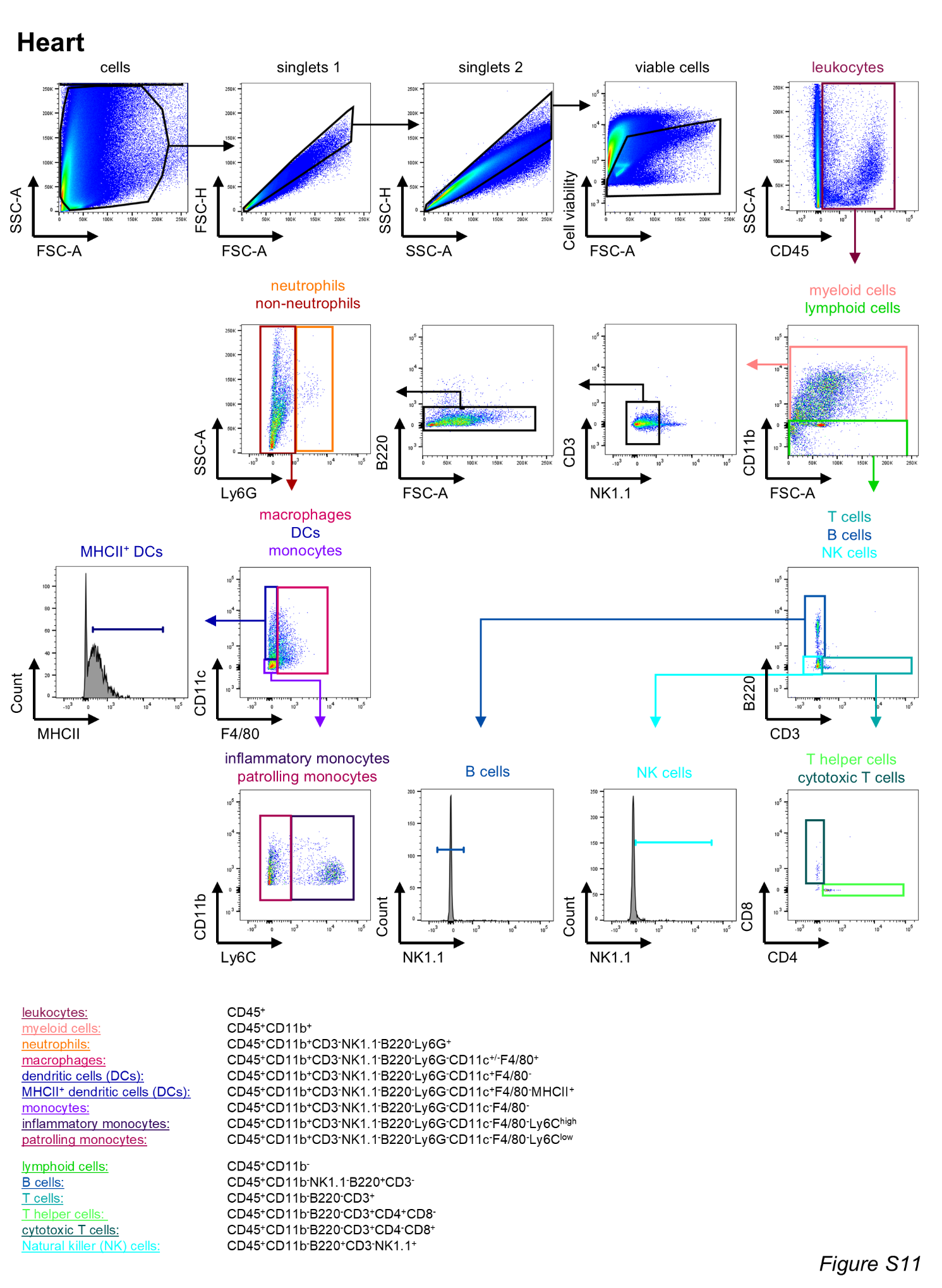


**Fig.S11 Representative gating strategy for quantifying cardiac immune cells during infection by flow cytometry.** To characterize immune cells from cardiac tissue, cells were stained with a panel of fluorochrome-conjugated antibodies targeting lymphoid and myeloid surface markers and analyzed by flow cytometry. Debris and cell doublets were first excluded using FSC-A/SSC-A, FSC-A/FSC-H, and SSC-A/SSC-H gating. Dead cells were then removed based on staining with viability dye. Subsequently, leukocytes were selected, and distinct myeloid and lymphoid subsets were identified according to specific marker combinations. The gating parameters for each immune cell population are described in detail below the representative gating plots


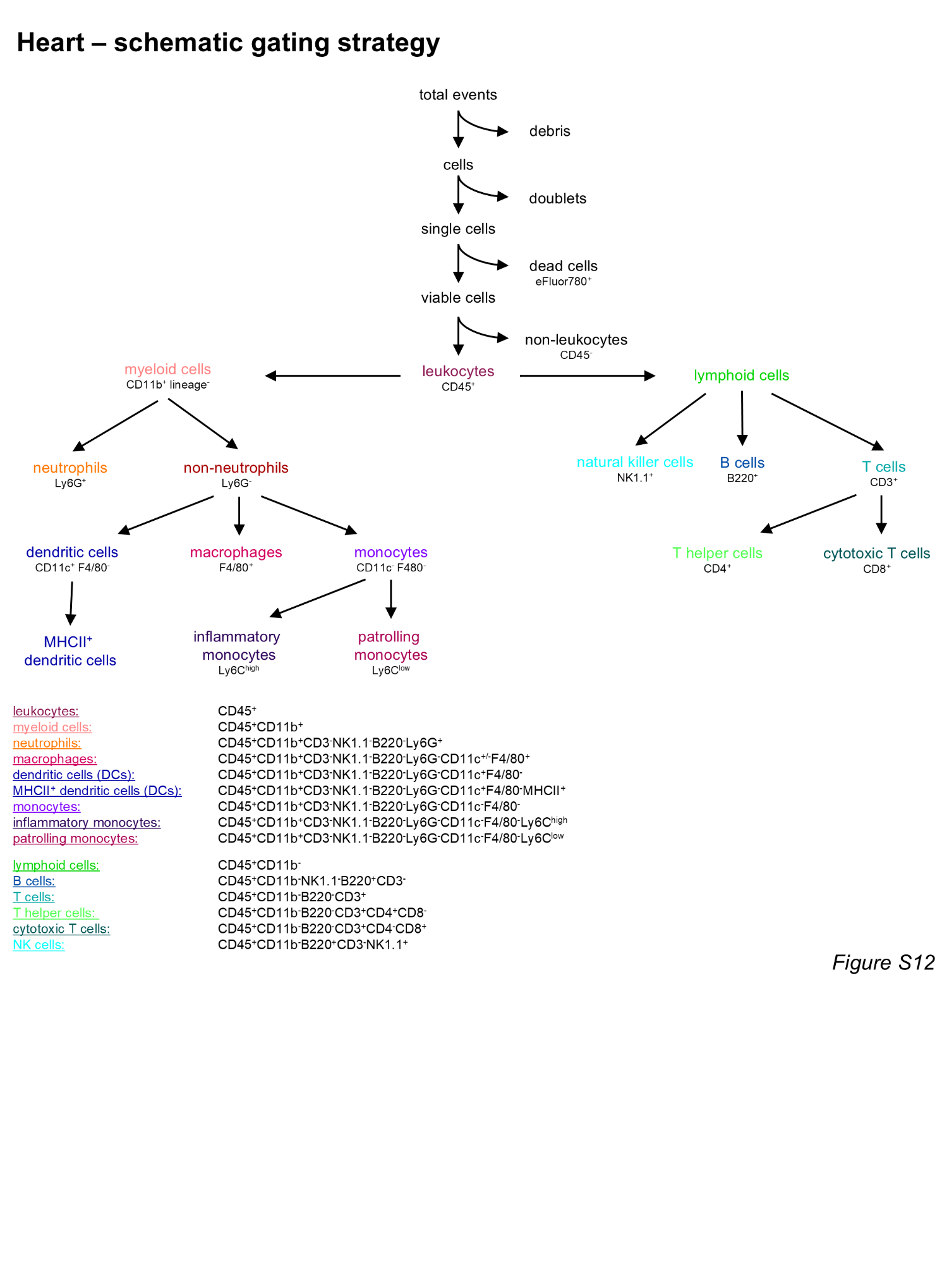


**Fig.S12 Schematic overview of the flow cytometry gating strategy for cardiac immune cells.** This scheme illustrates the gating strategy applied in the flow cytometry analysis summarizing the representative gating plot presented in Figure S6. The gating parameters for each immune cell population are indicated below the schematic overview

**Table S1. Flow cytometry antibody panels for immune cell quantification**. Table S1 lists all antibody-fluorochrome conjugates used for flow cytometric quantification of immune cells in heart and liver tissue, including clones, dilutions, suppliers, and catalogue numbers.

| **Antibodies used for flow cytometry of heart tissue** | | | | |
| --- | --- | --- | --- | --- |
| antibody - fluorochrome | clone | dilution | company | catalogue number |
| B220 - BUV395 | RA3-6B2 | 1:200 | BD | 563793 |
| CD8 - PB | 53-6.7 | 1:100 |  | 558106 |
| MHCII - FITC | AF6-120.1 | 1:150 |  | 553551 |
| CD3 - BUV737 | 145-2C11 | 1:200 |  | 612771 |
| CD11b - BV510 | M1/70 | 1:300 | Biolegend | 101245 |
| NK1.1 - PE | PK136 | 1:300 |  | 108707 |
| CD11c - PE dazzle | N418 | 1:200 |  | 117348 |
| Ly6G - BV605 | 1A8 | 1:400 |  | 127639 |
| F4/80 - APC | BM8 | 1:100 |  | 123116 |
| CD4 - PerCPCy5.5 | RM4-5 | 1:300 |  | 100539 |
| CD45.2 - BV711 | 104 | 1:200 |  | 109847 |
| Ly6C - PECy7 | HK1.4 | 1:400 |  | 128018 |
| **Antibodies used for flow cytometry of liver tissue (lymphoid panel)** | | | | |
| antibody - fluorochrome | clone | dilution | company | catalogue number |
| CD8 - PB | 53-6.7 | 1:100 | BD | 558106 |
| CD4 - V500 | RM4-5 | 1:100 |  | 560782 |
| CD45 - BV711 | 104 | 1:300 |  | 563685 |
| CD3 - BUV395 | 500A2 | 1:400 |  | 740221 |
| B220 - FITC | RA3-6B2 | 1:200 | Biolegend | 103206 |
| CD19 - PECy7 | 6D5 | 1:400 |  | 115519 |
| NK1.1 - PE-TR | PK136 | 1:300 |  | 108748 |
| CD69 - APC | H1.2F3 | 1:300 |  | 104513 |
| CD62L - AF700 | MEL-14 | 1:200 |  | 104426 |
| CD44 - PE | IM7 | 1:400 | Life Technologies | 12-0441-81 |
| **Antibodies used for flow cytometry of liver tissue (myeloid panel)** | | | | |
| antibody - fluorochrome | clone | dilution | company | catalogue number |
| MHCII - FITC | AF6-120.1 | 1:150 | BD | 553551 |
| TIM4 - BV421 | 21H12 | 1:300 |  | 742773 |
| CD86 - BUV395 | GL1 | 1:200 |  | 564199 |
| CD11b - BV510 | M1/70 | 1:300 | Biolegend | 101245 |
| Ly6G - BV605 | 1A8 | 1:400 |  | 127639 |
| CD169 - PerCPCy5.5 | 3D6.112 | 1:150 |  | 142409 |
| CD3 - PE | 145-2C11 | 1:300 |  | 100307 |
| B220 - PE | RA3-6B2 | 1:300 |  | 103208 |
| Ter119 - PE | TER-119 | 1:300 |  | 116208 |
| CD49b - PE | DX5 | 1:300 |  | 108908 |
| CD11c - PE-TR | N418 | 1:200 |  | 117348 |
| Ly6C - PECy7 | HK1.4 | 1:400 |  | 128018 |
| F4/80 - APC | BM8 | 1:100 |  | 123116 |
| CD45.2 - AF700 | 104 | 1:200 |  | 109822 |
